# Impedance Characterization and Modeling of Gold, Silver, and PEDOT:PSS Ultra-Thin Tattoo Electrodes for Wearable Bioelectronics

**DOI:** 10.3390/s25154568

**Published:** 2025-07-23

**Authors:** Antonello Mascia, Riccardo Collu, Nasreddine Makni, Mattia Concas, Massimo Barbaro, Piero Cosseddu

**Affiliations:** Department of Electrical and Electronics Engineering, University of Cagliari, Piazza D’Armi, 09123 Cagliari, Italy; riccardo.collu@unica.it (R.C.); nasreddine.makni@unica.it (N.M.); mattia.concas@unica.it (M.C.); massimo.barbaro@unica.it (M.B.)

**Keywords:** tattoo electrodes, epidermal electronics, PEDOT:PSS, skin–electrode impedance, modeling

## Abstract

This study presents a comprehensive evaluation and an equivalent circuit modeling of the skin–electrode impedance characteristics of three types of ultra-thin tattoo electrodes, all based on Parylene C nanofilms but with different active materials: Gold, Silver, and PEDOT:PSS. Their performance was compared to standard disposable Ag/AgCl electrodes. Impedance measurements were carried out on six human subjects under controlled conditions, assessing the frequency response in the range of 20 Hz to 1 kHz. For each subject, the impedance was recorded six times over one hour to investigate the stability and the temporal performance. The collected data were subsequently analyzed to model the electrical properties and interface behavior of each electrode type. The findings demonstrate that the tattoo electrodes offer impedance levels comparable to those of Ag/AgCl electrodes (in the order of tens of kΩ at 20 Hz), while providing additional benefits such as enhanced conformability, improved skin adhesion, and reduced skin irritation during use. Furthermore, the modeling of the skin–electrode interface through a more detailed equivalent circuit than the single time constant model enables a more detailed interface analysis and description, with fitting algorithm R^2^ scores of about 0.999 and 0.979 for the impedance magnitude and impedance phase, respectively. The proposed equivalent circuit offers valuable insights for optimizing electrode design, supporting the potential of Parylene C-based tattoo electrodes as promising alternatives for next-generation wearable bioelectronic applications.

## 1. Introduction

In recent years, epidermal sensors have emerged as rapidly advancing technologies in the field of epidermal electronics, driving the development of next-generation wearable devices. In this context, the quality of the interface between the human body and the tattooable sensors plays a crucial role, particularly in ensuring a reliable and stable long-term signal acquisition. As highlighted by a range of studies, tattoo electrodes are ultra-thin and conformable devices with particular advances in the field of biomonitoring, such as ECG [1,2,3], EMG [4,5,6], and more recently EEG [7,8,9] and EOG [10,11], where the accurate detection of biopotential signals is essential for both clinical and non-clinical health monitoring systems. In addition, tattoo electrodes can ensure a stable, imperceptible, and skin-irritation-free interface with the human body [12,13,14].

However, there remains a need for an in-depth study of the electro–skin interface, particularly in the context of tattoo electrodes. Unlike Ag/AgCl electrodes, which are pre-gelled, tattoo electrodes are considered a dry type, since they can be attached onto the skin without requiring specialized gels or adhesives [15,16].

Given these considerations, studying the impedance of the electrode–skin interface is crucial for enhancing the performance and reliability of next-generation epidermal electrodes. The electrode–skin interface displays complicated, frequency-dependent behavior, whereby the resistive and capacitive components change according to various factors, such as the skin hydration, electrode material, and contact conditions [17,18]. To provide a clearer understanding of these electrical properties, researchers have developed multiple equivalent circuit models, which have been essential for enhancing electrode design and refining methods of signal acquisition [19,20]. One of the simplest models used to analyze the impedance of the electrode–skin interface is the Single Parallel RC Model. However, because of the complicated composition of the skin, this model could be incapable of describing its impedance characteristics [21]. The single time constant model, indeed, represents one of the most employed equivalent circuits to describe the skin–electrode interface impedance [22]. More detailed approaches—for instance, the Montague model and several levels of the Tregear model [23]—involve multilayered resistance (R) and capacitance (C) components in series or parallel combinations, thereby modeling the physiological characteristics of various skin layers [24]. Subsequent advancements in impedance modeling have incorporated Fractional-Order Elements, specifically the Constant Phase Element (CPE), to introduce frequency-dependent impedance behavior to more accurately capture the non-ideal behavior of the interface [25]. The CPE has been extensively applied in well-established models, including those of Cole [26], Thomasset, Kirkup, and Lapicque, to examine the electrical activity of the electrode–skin interface [27].

For this characterization, impedance fitting, which is a computational algorithm-dependent approach, represents a key method for accurately estimating model parameters. The Nonlinear Least Squares (NLLS) approach, typically executed through the Levenberg–Marquardt algorithm [28], iteratively minimizes the difference between the observed and theoretical impedance spectra using least squares. NLLS fitting, nevertheless, is susceptible to a strong dependence on the initial parameter guess and possibly falls into local minima, where it loses effectiveness [29]. Alternatively, the Genetic Algorithm (GA) has also attracted significant interest because it can explore a broader solution space by evolving multiple candidate solutions through selection, crossover, and mutation. The GA has been demonstrated to model human skin impedance effectively, with reduced fitting errors compared to traditional methods [30], although its convergence rate is population-size-dependent [31]. In recent years, machine learning (ML) techniques have also been discovered to be an effective approach for impedance fitting and circuit parameter estimation. Unlike iterative curve-fitting techniques, ML models can capture complex correlations between impedance data and circuit parameters or even predict the physiological status. Zhu et al. collected impedance data from over 500 Nyquist curves and used ML tools to train models that could automatically select the most suitable equivalent circuit [32]. In addition, Zulueta et al.’s study proposed a novel unsupervised artificial neural network (ANN) training cost function to estimate equivalent circuit parameters from electrochemical impedance spectroscopy (EIS) data [33]. Although machine learning (ML)-based fitting greatly enhances the speed of the impedance analysis and minimizes the dependence on expert knowledge and experience, there are still difficulties in the creation of large-scale EIS databases and in addressing the computational cost of training large-scale models [34].

In this study, we evaluate the skin–electrode impedance of three types of ultra-thin tattoo electrodes based on Parylene C, employing Gold, Silver, and PEDOT:PSS as active materials and compare them to standard disposable wet Ag/AgCl electrodes. We employed four distinct circuit models, starting from the well-established but simple single time constant model to more complex circuits aimed at deeply describing the skin–electrode interface. Moreover, we additionally assessed the variations in electrical parameters throughout a one-hour experiment of the best-fitting circuit, aiming to identify which parameters most significantly influence changes in the contact impedance over time. From the analysis of the tattooable and the disposable electrodes, results highlight a comparable impedance response in the range of 20 Hz–1 kHz, even in one hour of measurement, while tattoo electrodes improve the device’s conformability and reduce skin irritation. Moreover, the impedance fitting procedure—comprising the data processing, circuit modeling, parameter estimation, and model validation—resulted in an accurate characterization of the impedance, as quantitatively assessed by the R^2^ score of both the magnitude and phase. Our proposed model not only provides a better representation and fit of the skin–electrode interface impedance compared to existing models, but it is also capable of accurately fitting both the magnitude and the phase responses. In addition, from the analysis of the model circuits’ parameter variations over time, it is possible to predict the interface variations and anticipate changes in the impedance behavior under diverse conditions, an aspect that holds significant importance for the future development of next-generation wearable and epidermal devices tailored to healthcare monitoring, diagnostics, and personalized medicine.

## 2. Materials and Methods

### 2.1. Tattoo Electrode Fabrication Process

In this study, three types of tattoo electrodes based on a Parylene C nanofilm were developed: (i) Gold (Au), (ii) Silver (Ag), and (iii) a composite made from two different formulations of Poly(3,4-ethylenedioxythiophene):polystyrene sulfonate (PEDOT:PSS) (Clevios PH1000, by Heraeus, Hanau, Germany)—one being a highly conductive aqueous dispersion and the other containing 2.58 wt% NMP—combined with ethylene glycol, with a final mixture composition of 50%, 30%, and 20% by volume of each component, respectively.

**Figure 1:**
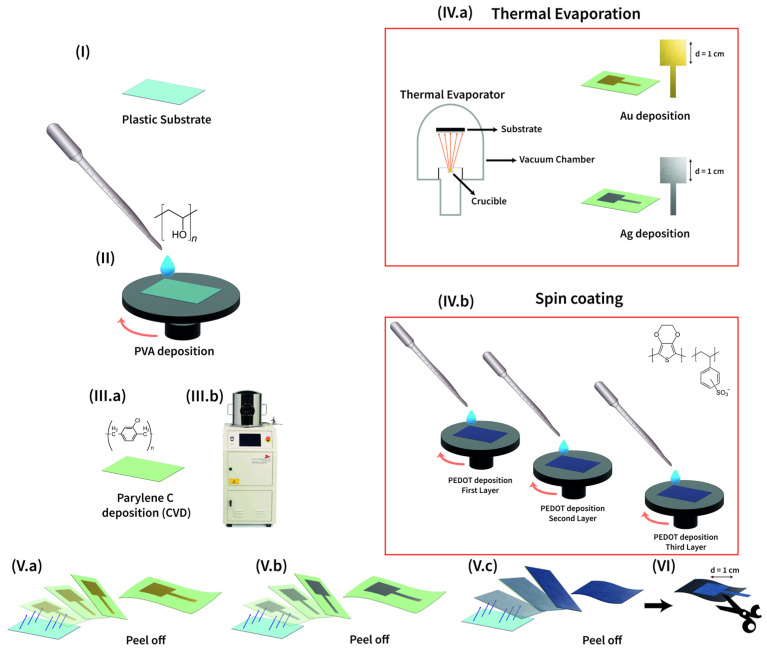
The Parylene C-based tattoo electrode fabrication process (**I**). The plastic carrier onto which (**II**) the PVA water solution was spin-coated. The Parylene C film was deposited through CVD (**III.a,b**). Gold and Silver electrodes were deposited via thermal evaporation using a shadow mask (**IV.a**), while PEDOT:PSS electrodes were solution processed (**IV.b**). Finally, electrodes were peeled off of the plastic carrier ((**V.a**) Au, (**V.b**) Ag, (**V.c**) PEDOT:PSS), and, additionally, PEDOT:PSS electrodes were cut to match the electrode dimensions (**VI**).

All the tattoo electrodes were fabricated starting from a Polyethylene Naphthalate (PEN) 125 µm thick substrate, sonicated with acetone, and cleaned with ethanol and deionized water (Figure 1I). On top of it, a 6% wt Polyvinyl Alcohol (PVA) solution in deionized water was spin-coated and then baked at 90 °C in an oven (Figure 1II). This first layer, 3 µm thick, acts as a sacrificial layer, whereas to realize a free-standing support for the conductive electrode, a sub-micrometric (600 nm) Parylene C film was deposited through Chemical Vapor Deposition (CVD), as reported in Figure 1(IIIa,b). Once treated in this way, the substrate is ready to host the electrode. Au and Ag were deposited through the thermal evaporation technique, with a shadow mask (active electrode area of 1 cm^2^) that avoids the need for any patterning (see Figure 1(IVa)). PEDOT:PSS-based solution was spin-coated on the whole substrate and then thermally annealed; in this way, three layers of mix were deposited (Figure 1(IVb)). Subsequently, the electrodes were peeled off from the plastic carrier (Figure 1(Va–c)) and additionally PEDOT:PSS-based ones were cut in the required shape to match the electrode dimensions (Figure 1VI).

### 2.2. Electrode–Skin Impedance Acquisition Set-Up

**Figure 2:**
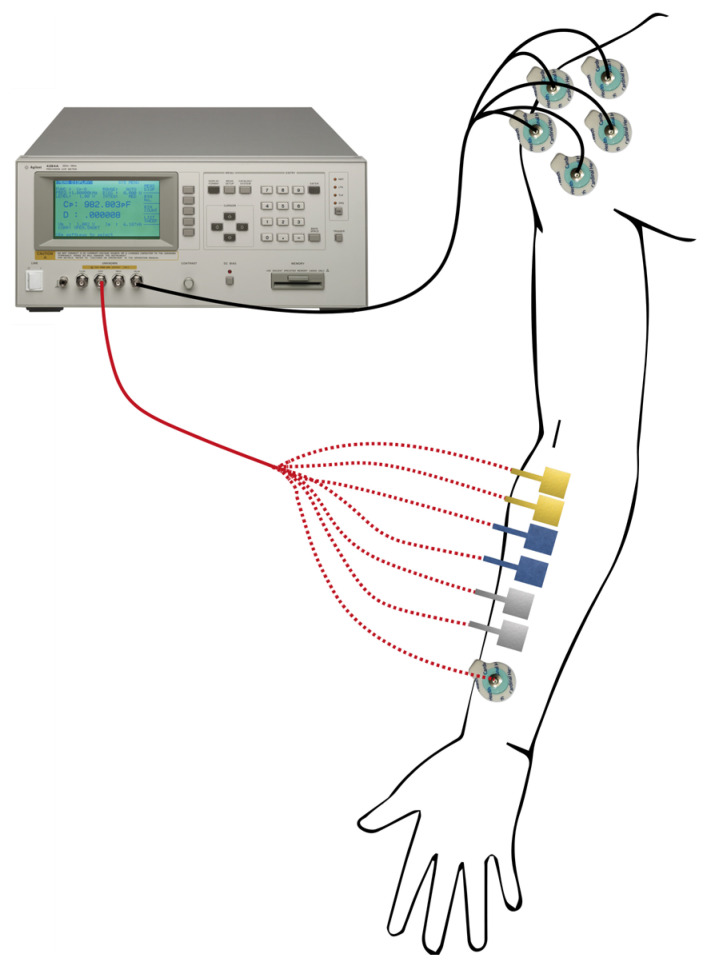
The skin–electrode impedance set-up. The tattoo electrodes, as well as the disposable Ag/AgCl electrode, were positioned on the subject’s forearm, and the impedance was acquired with respect to the parallel of five Ag/AgCl electrodes placed on the subject’s shoulder.

The Agilent 4284A precision LCR meter (Agilent Technologies Inc., Santa Clara, CA, USA) was used to evaluate the skin–electrode impedance. Two pairs of electrodes for each type were placed on the subject’s forearm, while a disposable Ag/AgCl electrode was employed as the gold standard for the comparison. The measurement set-up is shown in Figure 2. As we have done in previous studies [7,12,35,36], the obtained results were compared with impedance measurements from a set of five pre-gelled commercial electrodes in parallel. This set-up was designed to maximize the skin–tattoo electrode interface and simultaneously minimize the interface between the skin and the Ag/AgCl electrodes during data acquisition. This enables the acquisition of acquired data, which enables the acquisition of data that is related only to the tattoo electrode interface, while the parallel of five disposable electrodes is negligible.

### 2.3. Impedence Fitting Algorithm

Impedance fitting procedure was performed using MATLAB^®^ R2023b (The MathWorks Inc., Natick, MA, USA), and it involves preprocessing of the data, circuit modeling, parameter estimation, and model validation for accurate impedance characterization.

The raw data are first recorded in polar coordinates, i.e., magnitude $\left|Z\right|$ and phase θ, and then converted to Cartesian coordinates, i.e., real $Z^{\prime }$ and imaginary $Z^{\prime \prime }$ components, using the following equations:

(1) $$Z^{\prime }=\left|Z\right|cos\left(\theta \right),Z^{\prime \prime }=|Z|sin(\theta )$$

**Figure 3:**
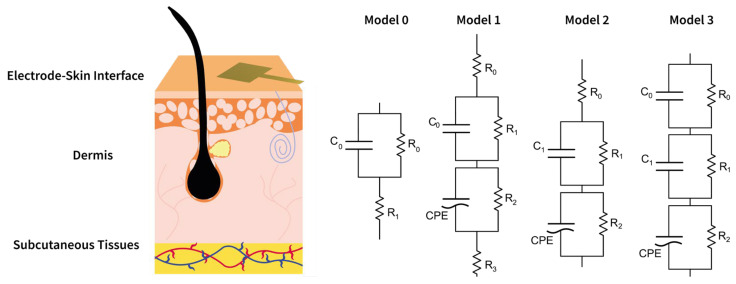
A schematic representation of the four different equivalent electrical circuit models used to describe the skin–electrode interface.

In order to identify the optimal equivalent circuit model (ECM), four different models—referred to as model 0, model 1, model 2, and model 3—were selected to represent the electrode–skin interface, as shown in Figure 3. Model 0 corresponds to the well-established single time constant model, whereas models 1, 2, and 3 offer increasingly detailed approaches for fitting and describing the skin–electrode interface. Particularly, the employed models comprise resistive (R), capacitive (C), and Constant Phase Elements (CPEs) to represent frequency-dependent behavior.

To characterize the electrical impedance response of the skin–electrode interface, we employed a nonlinear least squares optimization approach using MATLAB^®^’s *lsqnonlin* function (Optimization Toolbox) for each model. This function is optimized to solve nonlinear least squares (nonlinear data-fitting) problems and, particularly, this approach allows robust parameter estimation for a variety of equivalent electrical circuit models, commonly used in bioimpedance spectroscopy. The aim of the fitting is based on the comparison between the modeled complex impedance $\hat{Z}$(*f*) to the experimental measurements, separately evaluating both the magnitude ($\left|Z\right|$) and phase (θ) across all frequency points:

(2) $$residual=\left[w_{mod}\cdot \frac{\left|\hat{Z}\left(f\right)-Z(f)\right|}{\left|Z(f)\right|},w_{phi}\cdot \frac{arg(\hat{Z}\left(f\right))-arg(Z(f))}{\left|arg(Z(f))\right|}\right]$$

where $w_{mod}$ and $w_{phi}$ are user-defined weights (default: 0.5 each), enabling balanced sensitivity to magnitude and phase errors.

With the aim of mitigating local minima and simultaneously improving global convergence, a multi-start strategy was employed. The algorithm performs multiple fitting iterations (300 trials) using a custom initial condition vector. At each iteration, the *lsqnonlin* optimizer seeks the parameter vector that minimizes the Euclidean norm of the residual vector. This approach increases the likelihood of identifying the global minimum of the cost function, yielding more reliable and physically meaningful parameter estimates by reducing the dependence on initial conditions and avoiding convergence to suboptimal local minima.

Performance is quantified through the coefficient of determination ($R^{2}$), which is calculated separately for impedance magnitude and phase:

(3) $$R^{2}=1-\frac{\sum {\left(Z(f)-\hat{Z}\left(f\right)\right)}^{2}}{\sum {\left(Z(f)-\bar{Z}\right)}^{2}}$$

where Z(f) and $\hat{Z}\left(f\right)$ are the measured and fitted impedance values, respectively.

## 3. Results and Discussions

The impedance between the electrode and the skin is a key parameter that significantly affects the quality of the acquired bioelectrical signal. Usually, it has a complex frequency dependency. We measured the skin–electrode impedance magnitude of the electrodes in the range of 20 Hz to 1 kHz, which is the standard frequency range for biopotential applications.

**Figure 4:**
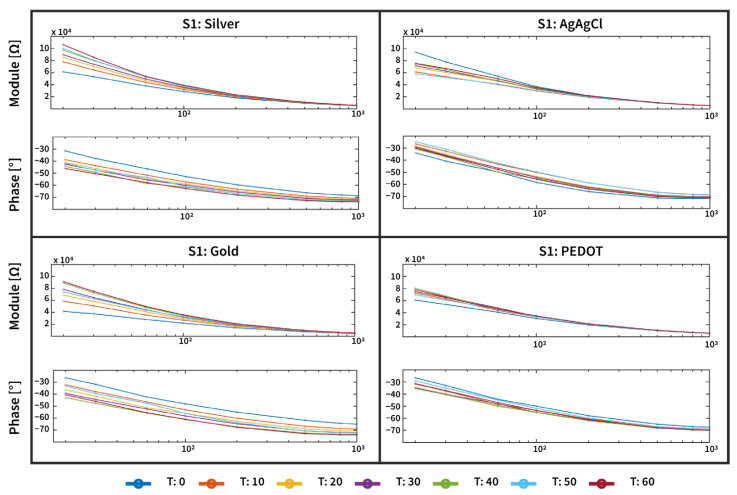
An example of the magnitude and phase angle response acquired over the 1 h experiment of the three types of tattoo electrodes (Silver, Gold, and PEDOT:PSS) and the disposable Ag/AgCl electrode employed for this study. The magnitude and phase response of the tattoo and disposable electrodes are comparable (in the order of tens of kohm at 20 Hz), thus enabling a good-quality biopotential signal acquisition for the epidermal system.

An example of the magnitude and phase angle responses of three types of tattoo electrodes, and for the disposable Ag/AgCl electrode used as a gold standard, is reported in Figure 4. As can be observed, the skin–electrode interface changes over time, and for the three dry tattoo electrodes, in particular, the impedance magnitude tends to increase, while the modulus of the standard Ag/AgCl slightly decreases. This different behavior can be attributed to the different categories of devices employed. In fact, while tattoo electrodes are dry electrodes, disposable Ag/AgCl electrodes are wet electrodes. Such devices are used as the standard for biopotential measurements due to their stable and low-impedance interface with the skin. However, they have some limitations; in particular, the gel employed to reduce the impedance can dry out over time, leading to a degraded signal quality, increased impedance, and poor long-term stability. Moreover, the need for skin preparation and adhesive gels can cause discomfort and even skin irritation during extended use. These drawbacks limit their suitability for long-term, wearable, or ambulatory monitoring applications [37]. However, these variations are still compatible with the necessary skin–electrode impedance values for biopotential acquisition applications, as already shown in [12], where tattoo electrodes were employed for an ECG acquisition over 9 h of measurement.

Therefore, the fitting for each model was performed separately at each time point to assess how the electrical parameters evolve throughout the measurement period. First, the obtained results are in the same range and are consistent with findings reported in the existing literature [19,20,38,39], regarding the electrical parameters in particular.

**Figure 5:**
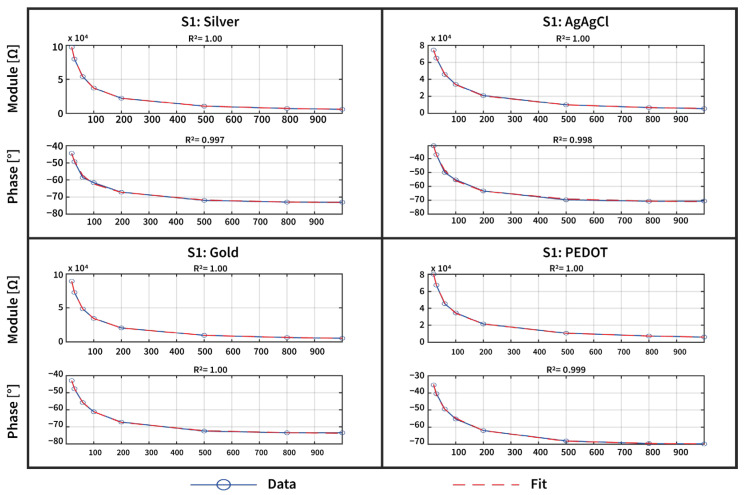
An example of the acquired impedance data (blue dots and line) and model 3 fit (red line) for one subject, shown for all three tattoo electrodes and the Ag/AgCl electrode in both the magnitude and phase angle. The close agreement between the measured data and the model fit, along with the high R^2^ scores achieved, quantitatively demonstrates the high-quality characterization of the skin–electrode impedance.

The coefficient of determination (R-squared, R^2^) was employed to quantitatively evaluate the fitting performance, as it measures the proportion of variance in the dependent variable that is explained by the regression model. This criterion facilitates the identification of the model yielding the optimal fit, as reported in the Supplementary Materials, where the electrical parameters for each fitting algorithm are reported. Moreover, the use of R^2^ ensures a robust and reproducible impedance fitting procedure, thereby improving the reliability of the circuit parameter estimation. In particular, results show that model 3 has the best fitting regarding both the magnitude and the phase of the complex impedance describing the skin–electrode interface of the tattoo electrodes, as shown in Figure 5.

Although models 0–2 effectively describe the contact impedance magnitude, with a good coefficient of determination (reported in the Supplementary Materials), model 3 better fits both the magnitude and phase of this complex impedance, with an average R^2^ of 0.999 for the impedance magnitude and 0.979 for the impedance phase for the four types of electrodes. This improved performance is attributed to the more sophisticated structure of model 3, which more effectively represents the frequency-dependent behavior of the skin–electrode interface.

**Figure 6:**
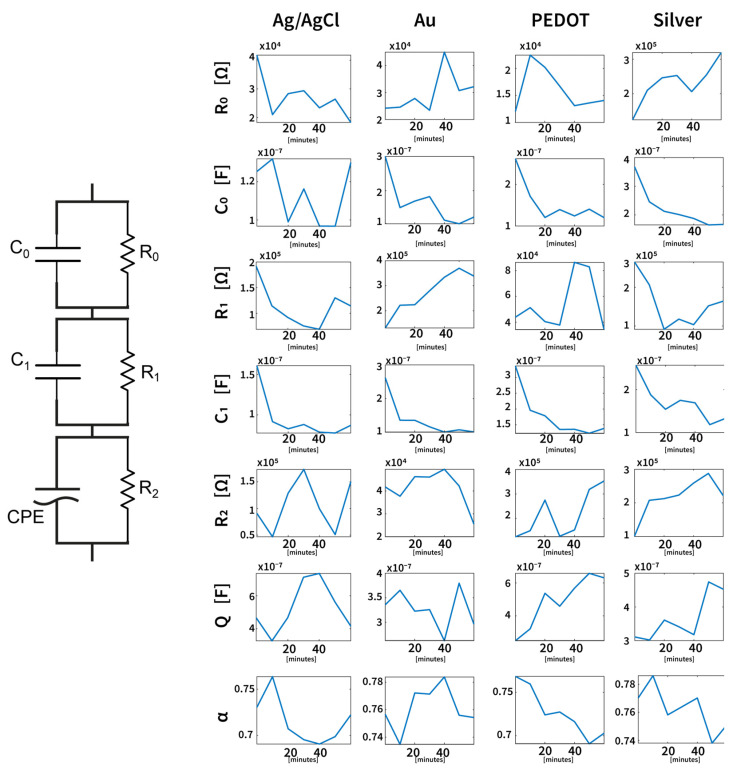
Median values (over six subjects) of the electrical parameters derived from model 3 and their variations across one hour of the experiment. Circuit elements have been grouped by the electrode type/material to better analyze time-dependent variations. The electrical parameters for the three types of tattoo electrodes show similar trends consistent with their dry electrode nature, while the Ag/AgCl electrode exhibits a different behavior characteristic of wet electrodes. Despite these differences, the proposed model is able to accurately capture and describe the impedance variations for all electrode types.

In addition to fitting the impedance at individual time points over one hour of experimentation for each subject, the models were also evaluated based on their ability to capture the variation in the impedance over time. As the skin–electrode interface properties change during the measurement period, accurately tracking these dynamic variations is crucial for both accurately describing the interface and improving the design of future electrodes. Among the models tested, model 3 demonstrated a superior performance in describing the temporal changes in both the impedance magnitude and phase. Its more detailed circuit representation allows it to adapt to the evolving electrical characteristics of the interface better than the other models, resulting in a more reliable fit across the entire duration of the experiment. These results highlight how the proposed model is capable of not only providing a static fitting but also modeling the time-dependent behavior of the skin–electrode interface. Figure 6 shows the values and their variation over time for the electrical parameters obtained throughout the model. The data represents the median parameter values measured across six subjects, illustrating how these electrical properties change during one hour of experimentation. This aspect is consistent with the skin–electrode impedance variation measured across time.

From the analysis of these parameters, it is possible to observe that the main difference between the standard disposable Ag/AgCl electrodes and the dry tattoo Silver, Gold, and PEDOT:PSS electrodes is remarkable in the first RC parallel segment, which is the one linked to the pure skin–electrode interface. In fact, while the parameter R_0_ increases for the tattoo electrodes (as well as the overall impedance as shown in the Bode diagram), the R_0_ related to Ag/AgCl electrodes tends to decrease. This aspect is compatible with the different types of electrodes; in fact, commercial wet electrodes are covered through an electrolyte gel, which is necessary to ensure a stable contact impedance [40]. Epidermal tattoo electrodes, on the other hand, do not require an electrolytic gel to ensure good contact impedance, but the ultra-low thickness guarantees not only a high resistance to mechanical deformation compatible with the characteristics of the skin but also a low-frequency contact impedance level compatible with the standard in biomedical applications [41,42], while simultaneously avoiding the use of adhesive layers that might provoke dermal irritations. Conversely, the parameter C_0_ related to tattoo electrodes tends to decrease over one hour of experimentation, approaching the same level of C_0_ for Ag/AgCl electrodes. Therefore, the overall increase in the impedance of the epidermal devices can be mainly connected to these variations in parameters in the modeled equivalent circuit. However, it is interesting to note that, differently from what already reported as the state of the art [43], there is no sweat effect that tends to change the interface by lowering the contact impedance in the tattoo electrode characterization, which is probably due to the controlled environment conditions in which the experiment was performed for all the subjects.

In addition to technical performance metrics that have been discussed above, user-centered factors such as comfort, wearability, and cost-effectiveness are critical for real-world applications, particularly in applications involving prolonged contact with human skin. The fabricated Parylene C-based tattoo electrodes belong to the category of epidermal electronics, where their flexibility, weight, unobtrusiveness, and ease of fabrication contribute to the overall usability of the device. Several recent studies have emphasized the importance of integrating these human-centric design principles into material development and system architectures [44,45]. Considering these aspects together with the proposed electrical circuit description of the skin–electrode interface from the early stages of design can significantly enhance the practicality and user acceptance of the final product.

## 4. Conclusions

In conclusion, this work presents a comprehensive evaluation of ultra-thin Parylene C-based tattoo electrodes with different active materials, namely Gold, Silver, and PEDOT:PSS, demonstrating an impedance performance comparable to that of standard Ag/AgCl electrodes. The tattoo electrodes’ impedance measurements across multiple subjects and over one hour revealed stable and reliable electrical behavior, with an improved conformability, skin adhesion, and comfort for the subjects due to the mechanical properties of the specific technology. Importantly, we proposed a detailed equivalent circuit model capable of accurately fitting both the magnitude and phase of the skin–electrode impedance. This model enabled us to extract and analyze the temporal variations in the electrical circuit parameters, offering deeper insight into the dynamic behavior of the skin–electrode interface. While Parylene C-based tattoo electrodes show great promise for wearable bioelectronics, classical limitations such as long-term adhesion, connections to standard acquisition electronic modules, and limited durability remain. These issues can be mitigated by ultra-thin device designs and cost-effective fabrication for disposability. Future work will focus on improving adhesion, mechanical robustness, and system integration to advance their practical deployment in long-term monitoring applications, and the possibility of modeling the skin–electrode interface can enable an improvement in the future tattoo electrode design and fabrications methods.

## Data Availability

The data that support the findings of this study are available from the corresponding authors upon request.

## Supplementary Materials

The following supporting information can be downloaded at: https://www.mdpi.com/article/10.3390/s25154568/s1, Supporting Information contains the extrapolated electrical parameters for all the three employed models and for the three types of materials (i.e., Gold, Silver, and PEDOT:PSS) and Ag/AgCl electrode used as reference. Table S1: Data Evaluation for Gold; Table S2: Data Evaluation for PEDOT:PSS; Table S3: Data Evaluation for Silver; Table S4: Data Evaluation for Ag/AgCl.
