# Supplementary material for: Impedance Characterization and Modeling of Gold, Silver, and PEDOT:PSS Ultra-Thin Tattoo Electrodes for Wearable Bioelectronics
Source: Sensors (Basel). 2025 Jul 23;25(15):4568. doi: 10.3390/s25154568 (PMC12349162; doi:10.3390/s25154568)
Supplement: Supplementary file 1 [file sensors-25-04568-s001.zip › DATA Supplementary/Table S1. Data_evaluation_Au.pdf]

Sub Model 0

0	R0	R1	C1	R^2	R^2 phi
1	1.41E+03	7.89E+04	5.91E-08	0.98362	-0.432
2	1.67E+03	6.19E+04	5.73E-08	0.98246	0.13626
3	1.34E+03	5.00E+04	6.93E-08	0.93746	0.59886
4	1.38E+03	1.14E+05	4.22E-08	0.99011	0.4742
5	1.05E+03	9.18E+04	6.49E-08	0.99018	-0.05698
6	1.47E+03	1.11E+05	5.88E-08	0.98894	-1.45659

10	R0	R1	C1	R^2	R^2 phi
1	1.24E+03	1.64E+05	4.35E-08	0.99234	0.11481
2	1.57E+03	9.29E+04	4.51E-08	0.99028	0.52788
3	1.30E+03	5.99E+04	5.51E-08	0.98824	0.70734
4	1.29E+03	1.43E+05	4.06E-08	0.9917	0.37775
5	1.02E+03	1.14E+05	5.92E-08	0.99209	0.05709
6	1.47E+03	1.30E+05	5.24E-08	0.99152	-0.99421

20	R0	R1	C1	R^2	R^2 phi
1	1.21E+03	1.85E+05	4.14E-08	0.99271	-0.00353
2	1.58E+03	9.52E+04	4.36E-08	0.99082	0.55157
3	1.24E+03	7.30E+04	5.23E-08	0.99239	0.65357
4	1.30E+03	1.81E+05	3.95E-08	0.99171	0.14038
5	9.98E+02	1.35E+05	5.83E-08	0.99299	-0.09185
6	1.43E+03	1.52E+05	5.12E-08	0.9919	-1.24811

30	R0	R1	C1	R^2	R^2 phi
1	1.15E+03	2.43E+05	3.92E-08	0.99391	-0.03343
2	1.59E+03	8.36E+04	4.20E-08	0.99144	0.71845
3	1.23E+03	8.47E+04	4.95E-08	0.992	0.62808
4	1.30E+03	1.94E+05	3.93E-08	0.99195	0.09805
5	1.01E+03	1.54E+05	5.44E-08	0.99381	-0.06693
6	1.40E+03	1.66E+05	5.03E-08	0.99212	-1.52785

40	R0	R1	C1	R^2	R^2 phi
1	1.15E+03	2.87E+05	3.64E-08	0.9946	-0.05051
2	1.60E+03	9.64E+04	4.09E-08	0.99266	0.63848
3	1.16E+03	1.02E+05	4.81E-08	0.99287	0.607
4	1.28E+03	1.91E+05	3.87E-08	0.99266	0.24086
5	1.09E+03	1.52E+05	5.05E-08	0.99496	0.02332
6	1.39E+03	1.83E+05	4.87E-08	0.99267	-1.65476

50	R0	R1	C1	R^2	R^2 phi
1	1.16E+03	3.16E+05	3.52E-08	0.99354	-0.28385
2	1.53E+03	1.05E+05	4.15E-08	0.99162	0.62876
3	1.22E+03	7.70E+04	4.64E-08	0.99133	0.8008
4	1.27E+03	1.88E+05	4.00E-08	0.99089	0.05372
5	9.90E+02	1.95E+05	5.36E-08	0.99457	-0.99977
6	1.40E+03	1.88E+05	4.69E-08	0.99226	-1.23256

60	R0	R1	C1	R^2	R^2 phi
1	1.34E+03	2.70E+05	2.96E-08	0.998	0.05352
2	1.57E+03	1.09E+05	3.98E-08	0.99133	0.64329
3	1.17E+03	1.03E+05	4.57E-08	0.99364	0.72322
4	1.33E+03	2.05E+05	3.84E-08	0.99211	0.07156
5	9.94E+02	1.96E+05	5.28E-08	0.99371	-0.89484
6	1.38E+03	1.99E+05	4.69E-08	0.99315	-1.69279

Model 1

0	R0	R1	C0	R2	Q	a	R3	R^2	R^2 phi
1	1.00E+01	6.77E+03	1.53E-07	1.99E+05	3.49E-07	0.73856	10	0.99993	0.99946
2	1.00E+01	5.36E+04	3.32E-07	5.64E+04	2.65E-07	0.75013	10	0.99995	0.99916
3	1.00E+01	9.90E+05	3.45E-06	5.36E+04	2.56E-07	0.77029	10	0.99991	0.99877
4	1.42E+02	1.52E+05	1.57E-07	7.44E+04	1.12E-07	0.84410	15.83095	0.99989	0.9985
5	2.06E+01	6.84E+03	2.43E-07	2.14E+05	2.73E-07	0.78366	20.62166	0.99999	0.99958
6	6.87E+01	3.28E+05	2.95E-07	1.26E+05	2.67E-07	0.75732	68.65825	0.99998	0.99566

10	R0	R1	C0	R2	Q	a	R3	R^2	R^2 phi
1	1.01E+01	2.94E+05	1.25E-07	1.02E+05	1.50E-07	0.82044	10.07724	0.99996	0.99824
2	2.21E+01	7.45E+04	2.58E-07	8.32E+04	1.57E-07	0.80025	22.10316	0.99998	0.99956
3	1.00E+01	3.17E+04	3.80E-07	5.45E+04	1.91E-07	0.80244	10	0.99969	0.9998
4	1.00E+01	2.06E+05	1.18E-07	8.10E+04	1.23E-07	0.83642	10	0.99997	0.99936
5	1.39E+01	1.26E+04	2.21E-07	3.02E+05	2.46E-07	0.79241	13.85985	0.99989	0.99745
6	1.60E+02	2.38E+05	1.52E-07	8.58E+04	2.22E-07	0.78634	160.17906	0.99995	0.99636

20	R0	R1	C0	R2	Q	a	R3	R^2	R^2 phi
1	1.01E+01	3.64E+05	1.01E-07	9.52E+04	1.44E-07	0.82749	10.12498	0.99999	0.99739
2	2.36E+01	7.76E+04	2.32E-07	8.13E+04	1.48E-07	0.80723	45.38681	0.99999	0.99959
3	1.00E+01	6.84E+04	6.26E-07	8.16E+04	1.62E-07	0.81436	10.00021	0.99988	0.99777
4	9.54E+01	2.81E+05	7.87E-08	6.33E+04	1.29E-07	0.84129	55.10855	0.99993	0.99962
5	5.87E+01	5.89E+03	3.11E-07	3.07E+05	1.73E-07	0.82789	58.70659	0.99983	0.99856
6	1.15E+02	2.58E+05	1.15E-07	7.64E+04	2.53E-07	0.77593	115.41829	1	0.9977

30	R0	R1	C0	R2	Q	a	R3	R^2	R^2 phi
1	1.00E+01	4.53E+05	7.40E-08	8.85E+04	1.62E-07	0.82507	10.00412	0.99952	0.99591
2	2.56E+01	4.91E+04	3.57E-07	8.11E+04	1.27E-07	0.82115	76.46061	0.99996	0.99936
3	2.99E+01	7.29E+04	2.47E-07	6.79E+04	1.50E-07	0.82780	29.88212	0.99998	0.99952
4	4.81E+01	3.01E+05	7.57E-08	6.65E+04	1.48E-07	0.82704	29.88896	0.99997	0.99279
5	1.00E+01	1.46E+04	2.47E-07	4.42E+05	1.84E-07	0.81886	55.10178	0.9999	0.99507
6	8.49E+01	3.10E+05	1.14E-07	8.83E+04	2.56E-07	0.77304	83.27563	0.99989	0.99793

40	R0	R1	C0	R2	Q	a	R3	R^2	R^2 phi
1	1.13E+01	1.00E+06	9.50E-08	1.81E+05	1.21E-07	0.84196	11.33741	0.9999	0.99282
2	1.00E+01	1.08E+05	4.76E-07	1.09E+05	1.28E-07	0.81394	10	0.99995	0.99781
3	4.63E+01	1.04E+05	2.00E-07	7.53E+04	1.40E-07	0.83701	28.84798	0.99999	0.99965
4	1.00E+01	3.00E+05	1.04E-07	1.12E+05	1.41E-07	0.82267	10	0.99989	0.96263
5	1.00E+01	1.96E+04	1.98E-07	4.21E+05	1.96E-07	0.80556	10	0.99929	0.98929
6	1.13E+02	3.72E+05	1.11E-07	1.03E+05	2.42E-07	0.77909	70.72821	0.99989	0.99826

50	R0	R1	C0	R2	Q	a	R3	R^2	R^2 phi
1	2.03E+02	9.92E+05	6.15E-08	9.37E+04	1.13E-07	0.87493	234.74644	0.99973	0.99715
2	4.21E+01	1.13E+04	1.51E-07	1.71E+05	1.76E-07	0.79582	42.10978	0.99993	0.99945
3	1.12E+02	1.15E+04	1.22E-07	1.02E+05	1.85E-07	0.82396	109.90021	0.99998	0.99889
4	1.00E+01	9.99E+05	1.49E-07	1.54E+05	1.23E-07	0.82983	10	0.99893	0.98204
5	1.76E+02	6.85E+03	2.34E-07	5.05E+05	1.24E-07	0.86728	144.0659	0.99996	0.9868
6	2.32E+02	4.24E+05	1.04E-07	9.16E+04	1.93E-07	0.80792	170.68453	0.99987	0.99676

60	R0	R1	C0	R2	Q	a	R3	R^2	R^2 phi
1	1.53E+02	9.64E+05	8.87E-08	1.67E+05	7.29E-08	0.87890	152.9987	0.99599	0.80678
2	3.02E+02	1.08E+05	1.11E-07	5.22E+04	1.00E-07	0.87061	301.50663	0.99993	0.9977
3	1.00E+01	8.41E+04	2.01E-07	7.72E+04	1.28E-07	0.84507	67.73514	0.99997	0.99952
4	1.00E+01	3.17E+05	7.30E-08	7.06E+04	1.57E-07	0.81796	10.001	0.99992	0.99057
5	1.23E+01	1.39E+04	2.88E-07	7.78E+05	1.61E-07	0.82745	34.92952	0.99999	0.99451
6	7.43E+01	3.83E+05	9.33E-08	8.86E+04	2.58E-07	0.77321	47.42008	0.99996	0.99643

Model 2

0	R0	R1	C0	R2	Q	a	R^2	R^2 phi
1	1.00E+01	6.73E+03	1.55E-07	1.99E+05	3.50E-07	0.73770	0.99993	0.99948
2	1.07E+01	5.30E+04	3.56E-07	5.89E+04	2.68E-07	0.74821	0.99996	0.99902
3	2.01E+02	1.41E+04	1.00E-06	4.44E+04	2.21E-07	0.79430	0.99977	0.99739
4	1.33E+02	1.53E+05	1.61E-07	7.64E+04	1.14E-07	0.84161	0.99989	0.99838
5	3.07E+01	6.85E+03	2.46E-07	2.15E+05	2.75E-07	0.78237	0.99999	0.99957
6	1.24E+02	4.08E+05	3.16E-07	1.33E+05	2.66E-07	0.75699	0.99998	0.9956

10	R0	R1	C0	R2	Q	a	R^2	R^2 phi
1	1.54E+01	2.98E+05	1.27E-07	1.04E+05	1.50E-07	0.82014	0.99996	0.99813
2	4.31E+01	7.44E+04	2.60E-07	8.36E+04	1.57E-07	0.80014	0.99998	0.99954
3	1.38E+01	5.00E+03	2.31E-07	8.73E+04	2.55E-07	0.77768	0.99980	0.99984
4	1.33E+01	2.03E+05	1.19E-07	8.17E+04	1.23E-07	0.83597	0.99997	0.99931
5	1.05E+01	1.30E+04	2.20E-07	3.07E+05	2.51E-07	0.78982	0.99989	0.9974
6	3.23E+02	2.38E+05	1.51E-07	8.55E+04	2.22E-07	0.78660	0.99995	0.99636

20	R0	R1	C0	R2	Q	a	R^2	R^2 phi
1	2.09E+01	3.87E+05	9.92E-08	9.32E+04	1.43E-07	0.82823	0.99999	0.99822
2	6.84E+01	7.76E+04	2.31E-07	8.13E+04	1.48E-07	0.80718	0.99999	0.99958
3	1.00E+01	5.83E+04	5.39E-07	7.82E+04	1.63E-07	0.81414	0.99987	0.998
4	1.54E+02	2.81E+05	7.87E-08	6.32E+04	1.29E-07	0.84163	0.99993	0.99961
5	1.11E+02	5.88E+03	3.14E-07	3.07E+05	1.74E-07	0.82720	0.99983	0.99856
6	2.64E+02	2.59E+05	1.20E-07	8.16E+04	2.48E-07	0.77723	0.99999	0.99714

30	R0	R1	C0	R2	Q	a	R^2	R^2 phi
1	1.89E+01	4.53E+05	7.39E-08	8.90E+04	1.63E-07	0.82406	0.99952	0.99569
2	1.58E+02	5.26E+04	3.21E-07	7.77E+04	1.24E-07	0.82508	0.99996	0.9995
3	2.23E+01	7.23E+04	2.44E-07	6.79E+04	1.53E-07	0.82483	0.99998	0.9996
4	7.73E+01	3.01E+05	7.57E-08	6.66E+04	1.48E-07	0.82715	0.99997	0.99279
5	8.32E+01	1.36E+04	2.52E-07	4.30E+05	1.80E-07	0.82204	0.99990	0.99521
6	1.93E+02	3.11E+05	1.12E-07	8.63E+04	2.53E-07	0.77534	0.99989	0.99834

40	R0	R1	C0	R2	Q	a	R^2	R^2 phi
1	5.67E+01	7.59E+05	7.69E-08	1.31E+05	1.33E-07	0.83963	0.99988	0.9958
2	1.76E+01	1.15E+05	4.86E-07	1.10E+05	1.28E-07	0.81369	0.99995	0.99781
3	6.54E+01	1.03E+05	2.00E-07	7.53E+04	1.41E-07	0.83619	0.99999	0.99965
4	1.05E+01	3.02E+05	1.06E-07	1.16E+05	1.40E-07	0.82241	0.99989	0.96303
5	2.98E+01	2.34E+04	1.78E-07	4.56E+05	2.10E-07	0.79969	0.99933	0.98713
6	1.85E+02	3.71E+05	1.10E-07	1.01E+05	2.43E-07	0.77898	0.99989	0.9983

50	R0	R1	C0	R2	Q	a	R^2	R^2 phi
1	4.41E+02	9.86E+05	6.15E-08	9.36E+04	1.12E-07	0.87518	0.99973	0.99705
2	9.21E+01	8.42E+04	2.02E-07	8.53E+04	1.32E-07	0.82077	0.99991	0.99945
3	2.60E+02	5.56E+04	1.82E-07	4.85E+04	1.15E-07	0.86414	0.99996	0.99915
4	2.53E+01	1.00E+06	1.49E-07	1.54E+05	1.24E-07	0.82837	0.99894	0.98123
5	3.66E+02	7.43E+03	2.16E-07	4.96E+05	1.21E-07	0.87261	0.99996	0.98724
6	4.02E+02	4.24E+05	1.04E-07	9.17E+04	1.93E-07	0.80790	0.99987	0.99677

60	R0	R1	C0	R2	Q	a	R^2	R^2 phi
1	9.24E+01	9.35E+05	9.32E-08	1.79E+05	7.73E-08	0.86741	0.99593	0.8097
2	6.10E+02	1.08E+05	1.11E-07	5.21E+04	9.96E-08	0.87128	0.99993	0.9977
3	5.99E+01	8.32E+04	2.06E-07	7.87E+04	1.29E-07	0.84365	0.99997	0.9995
4	1.98E+01	3.18E+05	7.29E-08	7.02E+04	1.57E-07	0.81805	0.99992	0.99056
5	1.44E+01	1.55E+04	2.81E-07	8.35E+05	1.68E-07	0.82257	0.99999	0.99414
6	1.45E+02	3.82E+05	9.46E-08	9.16E+04	2.57E-07	0.77361	0.99996	0.99634

Model 3

0	R0	C0	R1	C1	R2	Q	a	R^2	R^2 phi
1	3.50E+03	2.22E-07	1.04E+05	2.66E-07	6.91E+04	3.36E-07	0.74526	0.99999	0.99981
2	2.74E+03	3.04E-07	4.01E+04	1.00E-06	8.93E+04	3.42E-07	0.72703	0.99999	0.99988
3	2.22E+04	1.00E-06	2.01E+04	1.78E-07	2.10E+04	3.39E-07	0.75668	0.99987	0.99924
4	3.12E+04	9.91E-08	1.55E+05	1.32E-07	2.95E+04	2.79E-07	0.7757	0.99992	0.99791
5	3.09E+04	3.19E-07	1.31E+05	2.62E-07	4.18E+04	2.88E-07	0.78454	0.99999	0.99846
6	2.42E+04	4.27E-07	6.19E+05	3.02E-07	9.40E+04	3.49E-07	0.7307	0.99998	0.99493

10	R0	C0	R1	C1	R2	Q	a	R^2	R^2 phi
1	4.47E+04	1.28E-07	3.13E+05	1.11E-07	3.46E+04	2.57E-07	0.78862	0.99997	0.99912
2	8.65E+03	1.48E-07	1.89E+04	2.43E-07	1.64E+05	3.65E-07	0.72951	1	0.99991
3	1.52E+04	3.02E-07	6.98E+03	1.56E-07	7.59E+04	5.08E-07	0.72278	0.99985	0.99977
4	3.84E+04	1.30E-07	2.22E+05	1.19E-07	3.77E+04	1.83E-07	0.8145	0.99999	0.99969
5	2.46E+04	1.60E-07	1.92E+05	1.36E-07	2.33E+04	5.75E-07	0.73478	0.99991	0.99753
6	1.21E+04	2.83E-07	2.22E+05	1.41E-07	7.26E+04	4.49E-07	0.71443	0.99995	0.99545

20	R0	C0	R1	C1	R2	Q	a	R^2	R^2 phi
1	3.59E+04	1.68E-07	4.07E+05	9.57E-08	4.63E+04	2.19E-07	0.8014	0.99999	0.9978
2	5.69E+03	1.99E-07	1.25E+04	2.58E-07	1.65E+05	2.65E-07	0.75689	0.99999	0.99988
3	2.90E+04	1.31E-07	5.32E+03	1.56E-07	1.35E+05	7.27E-07	0.69836	0.99988	0.99922
4	2.78E+04	1.07E-07	2.85E+05	7.28E-08	2.34E+04	3.23E-07	0.77223	0.99995	0.99947
5	2.78E+04	2.77E-07	2.21E+05	1.41E-07	3.91E+04	3.48E-07	0.77663	0.99983	0.99757
6	8.65E+03	2.95E-07	2.24E+05	1.31E-07	1.09E+05	4.56E-07	0.71436	0.99998	0.99806

30	R0	C0	R1	C1	R2	Q	a	R^2	R^2 phi
1	6.81E+04	1.39E-07	5.41E+05	8.93E-08	4.61E+04	2.18E-07	0.80871	0.99961	0.99341
2	4.81E+03	1.94E-07	2.13E+04	1.37E-07	1.25E+05	3.63E-07	0.73337	0.99997	0.99952
3	1.40E+04	2.14E-07	4.49E+03	2.32E-07	1.42E+05	2.88E-07	0.7741	0.99997	0.99988
4	2.93E+04	1.13E-07	3.08E+05	6.98E-08	2.25E+04	3.26E-07	0.7714	0.99998	0.9925
5	2.34E+04	1.82E-07	2.80E+05	1.07E-07	3.23E+04	6.41E-07	0.72628	0.99991	0.99418
6	6.94E+03	3.71E-07	2.63E+05	1.34E-07	1.33E+05	3.76E-07	0.7322	0.99987	0.99791

40	R0	C0	R1	C1	R2	Q	a	R^2	R^2 phi
1	8.42E+04	1.10E-07	9.85E+05	8.36E-08	4.96E+04	2.47E-07	0.79833	0.99991	0.99544
2	6.78E+03	1.29E-07	4.07E+04	9.74E-08	2.11E+05	6.23E-07	0.6893	0.99995	0.99879
3	1.76E+04	2.06E-07	5.93E+03	2.21E-07	1.85E+05	2.62E-07	0.78384	1	0.99984
4	7.04E+04	8.36E-08	3.61E+05	1.01E-07	2.26E+04	2.39E-07	0.80062	0.99986	0.97191
5	4.49E+04	9.49E-08	2.78E+05	1.07E-07	1.03E+04	5.74E-07	0.74159	0.99937	0.98824
6	4.47E+03	6.50E-07	3.34E+05	1.36E-07	1.55E+05	2.93E-07	0.75385	0.99988	0.99617

50	R0	C0	R1	C1	R2	Q	a	R^2	R^2 phi
1	5.78E+04	7.04E-08	8.97E+05	5.56E-08	8.65E+03	6.31E-07	0.73616	0.9998	0.99504
2	1.35E+04	9.91E-08	5.02E+04	1.20E-07	2.04E+05	7.26E-07	0.67843	0.99998	0.99975
3	3.07E+04	2.77E-07	1.46E+04	1.12E-07	6.58E+04	3.80E-07	0.75601	0.99998	0.99909
4	9.32E+04	7.14E-08	1.00E+06	1.27E-07	2.08E+04	2.06E-07	0.81671	0.99922	0.99021
5	3.01E+04	4.03E-07	3.70E+05	9.87E-08	4.22E+04	3.31E-07	0.7833	0.99994	0.97642
6	2.00E+04	1.74E-07	3.67E+05	1.02E-07	9.31E+04	6.26E-07	0.69077	0.99991	0.99407

60	R0	C0	R1	C1	R2	Q	a	R^2	R^2 phi
1	1.04E+05	4.78E-08	7.95E+05	7.16E-08	1.34E+04	2.45E-07	0.80822	0.99618	0.80685
2	2.76E+04	7.65E-08	1.13E+05	9.91E-08	1.76E+04	6.18E-07	0.70157	0.99994	0.99807
3	4.29E+04	1.54E-07	1.53E+04	1.14E-07	1.55E+05	5.46E-07	0.72756	0.99999	0.99954
4	3.14E+04	1.19E-07	3.29E+05	6.80E-08	2.34E+04	2.77E-07	0.78536	0.99992	0.9914
5	3.22E+04	2.08E-07	5.46E+05	1.01E-07	3.76E+04	4.87E-07	0.74987	0.99999	0.99543
6	4.07E+03	5.44E-07	3.39E+05	1.20E-07	1.58E+05	2.96E-07	0.75433	0.99996	0.99556
