# Supplementary material for: Impedance Characterization and Modeling of Gold, Silver, and PEDOT:PSS Ultra-Thin Tattoo Electrodes for Wearable Bioelectronics
Source: Sensors (Basel). 2025 Jul 23;25(15):4568. doi: 10.3390/s25154568 (PMC12349162; doi:10.3390/s25154568)
Supplement: Supplementary file 1 [file sensors-25-04568-s001.zip › DATA Supplementary/Table S2. Data_evaluation_Pedot.pdf]

Sub Model 0

0	R0	R1	C1	R^2	R^2 phi
1	1.40E+03	1.32E+05	4.68E-08	0.99215	0.1865
2	1.48E+03	6.18E+04	6.75E-08	0.98835	0.10974
3	1.67E+03	6.01E+04	4.55E-08	0.98944	0.80862
4	1.24E+03	1.34E+05	4.80E-08	0.99213	0.23824
5	9.32E+02	1.01E+05	7.10E-08	0.99131	-0.3268
6	1.07E+03	9.13E+04	7.09E-08	0.99347	0.08082

10	R0	R1	C1	R^2	R^2 phi
1	1.37E+03	1.37E+05	4.69E-08	0.99276	0.12707
2	1.49E+03	6.47E+04	6.23E-08	0.9897	0.33267
3	1.59E+03	7.42E+04	4.53E-08	0.98896	0.73845
4	1.25E+03	1.46E+05	4.40E-08	0.99387	0.44674
5	9.17E+02	1.17E+05	6.46E-08	0.99332	0.01712
6	1.07E+03	9.51E+04	6.70E-08	0.99409	0.2652

20	R0	R1	C1	R^2	R^2 phi
1	1.39E+03	1.40E+05	4.54E-08	0.99272	0.15498
2	1.49E+03	6.46E+04	6.19E-08	0.98978	0.34494
3	1.58E+03	8.07E+04	4.50E-08	0.9903	0.6669
4	1.24E+03	1.57E+05	4.39E-08	0.99346	0.35596
5	9.13E+02	1.30E+05	6.28E-08	0.99451	0.00747
6	1.04E+03	1.23E+05	6.42E-08	0.9942	-0.01722

30	R0	R1	C1	R^2	R^2 phi
1	1.38E+03	1.43E+05	4.45E-08	0.99339	0.31199
2	1.50E+03	6.76E+04	5.79E-08	0.99042	0.47503
3	1.60E+03	8.09E+04	4.40E-08	0.99191	0.66834
4	1.28E+03	1.68E+05	4.12E-08	0.99392	0.37793
5	9.06E+02	1.46E+05	6.13E-08	0.99509	0.01974
6	1.04E+03	1.35E+05	6.24E-08	0.99427	-0.15151

40	R0	R1	C1	R^2	R^2 phi
1	1.39E+03	1.48E+05	4.30E-08	0.99402	0.35815
2	1.47E+03	6.62E+04	5.68E-08	0.98909	0.53481
3	1.55E+03	8.41E+04	4.51E-08	0.98826	0.66105
4	1.26E+03	1.79E+05	4.12E-08	0.99441	0.39536
5	9.22E+02	1.60E+05	5.96E-08	0.99549	-0.15448
6	1.05E+03	1.46E+05	6.10E-08	0.99501	-0.17646

50	R0	R1	C1	R^2	R^2 phi
1	1.40E+03	1.48E+05	4.26E-08	0.99413	0.43587
2	1.49E+03	7.16E+04	5.56E-08	0.98982	0.50762
3	1.63E+03	7.00E+04	4.22E-08	0.99196	0.79668
4	1.26E+03	1.81E+05	4.07E-08	0.99461	0.43616
5	9.32E+02	1.76E+05	5.89E-08	0.99523	-0.32532
6	1.06E+03	1.47E+05	5.97E-08	0.99507	-0.05081

60	R0	R1	C1	R^2	R^2 phi
1	1.41E+03	1.47E+05	4.19E-08	0.9944	0.50258
2	1.51E+03	6.85E+04	5.45E-08	0.99024	0.59564
3	1.57E+03	7.66E+04	4.28E-08	0.99031	0.77163
4	1.28E+03	1.90E+05	3.94E-08	0.9951	0.3986
5	9.40E+02	1.83E+05	5.88E-08	0.9948	-0.77209
6	1.04E+03	1.55E+05	5.97E-08	0.99494	-0.1789

Model 1

0	R0	R1	C0	R2	Q	a	R3	R^2	R^2 phi
1	1.18E+02	1.70E+05	1.39E-07	8.12E+04	1.68E-07	0.81351	122.88216	0.99999	0.99936
2	1.12E+02	3.48E+04	5.84E-07	7.35E+04	3.17E-07	0.75183	113.90626	0.99999	0.99973
3	1.00E+01	7.06E+04	2.89E-06	7.70E+04	1.59E-07	0.79492	10.01258	0.99993	0.99885
4	3.84E+01	1.86E+05	1.39E-07	8.11E+04	1.70E-07	0.81575	38.3852	0.99998	0.999
5	1.05E+02	2.51E+05	2.30E-07	7.20E+04	2.43E-07	0.8154	105.38936	0.99998	0.99605
6	2.21E+02	5.64E+03	3.34E-07	1.74E+05	2.26E-07	0.82434	220.56758	0.99999	0.99929

10	R0	R1	C0	R2	Q	a	R3	R^2	R^2 phi
1	5.97E+01	1.94E+05	1.67E-07	1.05E+05	1.69E-07	0.80698	61.90242	0.99995	0.99804
2	1.82E+02	4.31E+04	3.76E-07	6.17E+04	2.56E-07	0.77868	178.93665	0.99999	0.99994
3	1.00E+01	2.88E+04	4.08E-07	7.60E+04	1.61E-07	0.79826	10.00091	0.99974	0.99899
4	1.06E+02	1.99E+05	1.49E-07	1.01E+05	1.34E-07	0.83966	105.7145	0.99996	0.99744
5	1.70E+01	1.67E+04	2.16E-07	3.61E+05	2.63E-07	0.80384	107.97445	0.99997	0.99499
6	2.25E+02	5.05E+03	3.31E-07	1.68E+05	1.95E-07	0.8379	225.32911	0.99998	0.99984

20	R0	R1	C0	R2	Q	a	R3	R^2	R^2 phi
1	1.08E+02	2.12E+05	1.56E-07	1.01E+05	1.53E-07	0.81847	112.03175	0.99967	0.98792
2	1.62E+02	4.18E+04	3.88E-07	6.27E+04	2.55E-07	0.7765	157.36075	0.99997	0.99984
3	1.00E+01	4.15E+04	4.00E-07	8.37E+04	1.57E-07	0.79964	10	0.99999	0.99874
4	9.28E+01	9.33E+03	1.84E-07	2.97E+05	1.25E-07	0.843	121.06795	0.99976	0.99863
5	2.50E+02	8.46E+03	2.55E-07	2.84E+05	1.68E-07	0.8545	74.7722	0.99999	0.99842
6	1.55E+02	1.39E+04	2.79E-07	3.04E+05	2.28E-07	0.81534	154.52147	0.99999	0.99665

30	R0	R1	C0	R2	Q	a	R3	R^2	R^2 phi
1	1.13E+02	1.92E+05	1.55E-07	1.04E+05	1.47E-07	0.82423	117.88501	0.99997	0.99945
2	1.53E+02	3.82E+04	5.07E-07	7.33E+04	2.20E-07	0.78693	148.27694	0.99999	0.99986
3	1.27E+01	4.83E+04	5.19E-07	8.97E+04	1.46E-07	0.80506	12.70411	0.99985	0.99895
4	2.14E+02	1.46E+04	1.29E-07	3.15E+05	1.13E-07	0.86018	218.08132	0.99979	0.99838
5	1.19E+02	1.18E+04	3.37E-07	3.65E+05	1.83E-07	0.83485	38.61868	0.99993	0.99706
6	1.87E+02	1.03E+04	3.10E-07	3.28E+05	1.88E-07	0.83421	186.97238	0.99994	0.99835

40	R0	R1	C0	R2	Q	a	R3	R^2	R^2 phi
1	9.82E+01	1.78E+05	1.28E-07	9.24E+04	1.49E-07	0.82418	133.96254	0.99997	0.99891
2	1.39E+02	4.00E+03	3.24E-07	1.05E+05	2.58E-07	0.77312	138.19558	0.99989	0.99987
3	1.42E+02	6.51E+04	1.60E-07	5.25E+04	1.50E-07	0.81764	40.96971	0.99977	0.99967
4	1.75E+02	9.86E+03	1.63E-07	3.20E+05	1.03E-07	0.86467	182.41491	0.99994	0.99949
5	2.29E+02	8.28E+03	3.23E-07	3.75E+05	1.48E-07	0.85997	81.8374	0.99999	0.99553
6	2.47E+02	5.81E+03	3.47E-07	2.98E+05	1.47E-07	0.86251	249.04161	0.99993	0.99841

50	R0	R1	C0	R2	Q	a	R3	R^2	R^2 phi
1	8.83E+01	1.65E+05	1.48E-07	1.08E+05	1.42E-07	0.8257	120.14464	0.99997	0.99811
2	1.46E+02	3.99E+03	3.09E-07	1.15E+05	2.38E-07	0.78095	145.53903	0.99997	0.99987
3	1.53E+01	2.79E+04	7.63E-07	7.80E+04	1.28E-07	0.81799	76.15943	0.99976	0.99952
4	1.00E+01	1.61E+04	2.05E-07	3.71E+05	1.29E-07	0.82846	10	0.99956	0.99803
5	2.48E+02	6.74E+03	3.77E-07	4.11E+05	1.36E-07	0.86789	99.28423	0.99996	0.99821
6	2.51E+02	9.10E+03	2.94E-07	3.14E+05	1.53E-07	0.85851	247.94966	0.99999	0.99943

60	R0	R1	C0	R2	Q	a	R3	R^2	R^2 phi
1	1.77E+02	9.59E+03	1.82E-07	2.49E+05	1.21E-07	0.84381	194.41265	0.99995	0.9998
2	1.02E+02	4.70E+03	3.22E-07	1.06E+05	2.50E-07	0.77206	101.17005	0.99996	0.99985
3	4.87E+01	7.20E+03	1.76E-07	1.05E+05	1.82E-07	0.79421	46.22925	0.9999	0.99986
4	4.15E+01	1.95E+04	1.63E-07	4.09E+05	1.27E-07	0.83115	42.90904	0.99995	0.99774
5	2.12E+02	8.09E+03	3.04E-07	4.74E+05	1.36E-07	0.86927	176.13524	0.99991	0.99878
6	1.30E+02	2.87E+04	2.08E-07	5.58E+05	2.41E-07	0.80793	130.61239	0.99997	0.9922

Model 2

0	R0	R1	C0	R2	Q	a	R^2	R^2 phi
1	2.67E+02	1.71E+05	1.34E-07	7.76E+04	1.66E-07	0.81578	0.99999	0.99937
2	2.86E+02	3.78E+04	5.34E-07	6.99E+04	3.05E-07	0.75813	0.99999	0.99962
3	1.83E+02	2.01E+04	1.00E-06	6.83E+04	1.45E-07	0.81026	0.99994	0.99798
4	1.07E+02	1.89E+05	1.36E-07	7.83E+04	1.68E-07	0.81829	0.99998	0.99924
5	1.76E+02	2.56E+05	2.36E-07	7.43E+04	2.48E-07	0.81128	0.99998	0.99641
6	4.08E+02	1.07E+05	1.92E-07	5.22E+04	2.69E-07	0.81334	0.99999	0.99829

10	R0	R1	C0	R2	Q	a	R^2	R^2 phi
1	1.12E+02	1.95E+05	1.71E-07	1.07E+05	1.69E-07	0.8064	0.99995	0.99801
2	3.60E+02	4.30E+04	3.77E-07	6.19E+04	2.56E-07	0.77865	0.99999	0.99994
3	4.28E+01	3.71E+03	2.87E-07	1.06E+05	1.82E-07	0.78898	0.9998	0.99924
4	2.37E+02	1.97E+05	1.40E-07	9.48E+04	1.34E-07	0.84136	0.99996	0.99726
5	2.59E+02	2.82E+05	2.02E-07	7.93E+04	2.00E-07	0.83814	0.99996	0.99711
6	3.65E+02	1.03E+05	1.79E-07	5.42E+04	2.60E-07	0.81399	0.99996	0.99915

20	R0	R1	C0	R2	Q	a	R^2	R^2 phi
1	2.20E+02	2.11E+05	1.56E-07	1.01E+05	1.53E-07	0.81852	0.99967	0.98792
2	3.02E+02	4.06E+04	4.09E-07	6.45E+04	2.57E-07	0.77467	0.99998	0.99982
3	1.03E+01	6.81E+03	2.21E-07	1.23E+05	2.08E-07	0.77572	0.99997	0.99852
4	2.16E+01	1.97E+05	1.18E-07	9.17E+04	1.65E-07	0.81605	0.9997	0.99869
5	3.21E+02	2.04E+05	1.22E-07	4.63E+04	2.35E-07	0.83799	0.99999	0.99785
6	4.40E+02	1.98E+05	1.82E-07	7.75E+04	2.13E-07	0.83468	0.99999	0.99768

30	R0	R1	C0	R2	Q	a	R^2	R^2 phi
1	2.40E+02	1.92E+05	1.54E-07	1.03E+05	1.46E-07	0.8249	0.99997	0.99946
2	2.95E+02	3.77E+04	5.25E-07	7.43E+04	2.20E-07	0.78636	0.99999	0.99986
3	1.70E+01	4.97E+04	5.66E-07	9.17E+04	1.46E-07	0.80414	0.99985	0.99891
4	5.29E+02	2.30E+05	7.92E-08	5.36E+04	1.21E-07	0.87058	0.99985	0.99899
5	2.32E+02	2.51E+05	1.57E-07	8.67E+04	2.21E-07	0.83162	0.99995	0.99805
6	4.77E+02	2.18E+05	1.35E-07	6.04E+04	2.32E-07	0.83406	0.99993	0.99885

40	R0	R1	C0	R2	Q	a	R^2	R^2 phi
1	2.41E+02	1.78E+05	1.24E-07	8.92E+04	1.50E-07	0.82447	0.99997	0.99901
2	1.79E+02	3.19E+04	4.90E-07	7.14E+04	2.29E-07	0.77894	0.99986	0.99973
3	4.96E+01	6.28E+03	1.85E-07	1.28E+05	1.92E-07	0.78696	0.9998	0.99965
4	2.53E+02	2.32E+05	7.89E-08	6.10E+04	1.58E-07	0.83558	0.9999	0.99963
5	3.31E+02	2.71E+05	1.11E-07	5.65E+04	2.45E-07	0.83386	0.99999	0.99406
6	4.49E+02	2.16E+05	1.14E-07	5.17E+04	2.69E-07	0.82266	0.99991	0.99859

50	R0	R1	C0	R2	Q	a	R^2	R^2 phi
1	1.97E+02	1.65E+05	1.50E-07	1.08E+05	1.42E-07	0.82483	0.99997	0.99806
2	2.39E+02	3.91E+03	3.36E-07	1.16E+05	2.43E-07	0.77581	0.99997	0.99987
3	1.00E+01	1.26E+04	1.39E-07	9.20E+04	2.51E-07	0.76063	0.99964	0.99918
4	1.75E+01	2.70E+05	1.88E-07	1.70E+05	1.22E-07	0.83495	0.99967	0.99828
5	3.94E+02	3.00E+05	9.54E-08	4.52E+04	2.68E-07	0.83432	0.99996	0.99821
6	5.54E+02	2.26E+05	1.15E-07	5.27E+04	2.24E-07	0.84451	1	0.99926

60	R0	R1	C0	R2	Q	a	R^2	R^2 phi
1	2.29E+02	1.54E+05	1.18E-07	8.60E+04	1.49E-07	0.82552	0.99994	0.99906
2	1.89E+02	4.68E+03	3.28E-07	1.06E+05	2.51E-07	0.77078	0.99996	0.99984
3	3.67E+01	7.32E+03	1.83E-07	1.06E+05	1.88E-07	0.78886	0.9999	0.99984
4	1.22E+02	2.62E+05	1.02E-07	1.07E+05	1.36E-07	0.8341	0.99997	0.99783
5	4.55E+02	3.43E+05	9.16E-08	4.10E+04	2.48E-07	0.84482	0.99993	0.99887
6	4.31E+02	5.06E+05	2.20E-07	1.32E+05	1.75E-07	0.84637	0.99999	0.99457

Model 3

0	R0	C0	R1	C1	R2	Q	a	R^2	R^2 phi
1	1.01E+04	2.36E-07	8.89E+04	3.36E-07	1.78E+05	2.36E-07	0.7721	0.99999	0.99897
2	4.98E+03	3.22E-07	1.56E+04	4.38E-07	1.12E+05	7.14E-07	0.67083	0.99999	0.9998
3	2.55E+04	1.42E-07	2.49E+04	6.27E-07	3.39E+04	2.45E-07	0.76833	0.99992	0.99894
4	1.18E+05	2.62E-07	1.13E+04	1.99E-07	1.57E+05	2.49E-07	0.77947	0.99998	0.9995
5	1.21E+04	2.95E-07	2.38E+05	3.61E-07	1.25E+05	4.55E-07	0.74913	0.99998	0.99397
6	8.61E+03	2.92E-07	4.38E+04	2.62E-07	1.81E+05	7.46E-07	0.69826	0.99998	0.99549

10	R0	C0	R1	C1	R2	Q	a	R^2	R^2 phi
1	8.14E+03	1.76E-07	5.12E+04	1.40E-07	6.30E+05	4.48E-07	0.72237	0.99999	0.99892
2	3.16E+02	2.47E-09	3.91E+04	4.54E-07	6.80E+04	2.59E-07	0.77442	0.99999	0.99988
3	3.81E+04	1.43E-07	3.67E+04	7.28E-07	4.02E+04	2.07E-07	0.78435	0.99982	0.99935
4	1.24E+05	2.03E-07	2.38E+04	1.36E-07	1.50E+05	3.21E-07	0.75952	0.99998	0.99554
5	2.24E+04	1.71E-07	1.45E+05	2.57E-07	1.67E+05	6.98E-07	0.71782	0.99998	0.99293
6	8.36E+03	2.65E-07	5.35E+04	1.96E-07	1.77E+05	8.66E-07	0.68732	0.99997	0.99759

20	R0	C0	R1	C1	R2	Q	a	R^2	R^2 phi
1	2.02E+04	1.21E-07	6.85E+04	1.65E-07	6.21E+05	5.91E-07	0.69552	0.99968	0.98284
2	3.02E+02	1.24E-09	4.04E+04	4.17E-07	6.52E+04	2.57E-07	0.77502	0.99998	0.99967
3	7.31E+03	1.56E-07	2.05E+04	2.10E-07	1.15E+05	4.02E-07	0.72401	0.99999	0.99908
4	8.72E+04	1.05E-07	1.17E+04	1.33E-07	7.05E+05	5.37E-07	0.72076	0.99985	0.99817
5	1.23E+04	2.14E-07	9.59E+04	1.79E-07	2.75E+05	6.57E-07	0.72635	0.99998	0.99696
6	2.14E+04	1.86E-07	1.01E+05	2.13E-07	2.87E+05	9.52E-07	0.67979	0.99999	0.99427

30	R0	C0	R1	C1	R2	Q	a	R^2	R^2 phi
1	1.39E+04	1.39E-07	6.85E+04	1.25E-07	8.45E+05	5.79E-07	0.70097	0.99999	0.99886
2	2.96E+02	1.00E-09	3.77E+04	5.24E-07	7.42E+04	2.21E-07	0.78643	0.99999	0.99986
3	3.42E+03	2.20E-07	2.13E+04	1.35E-07	1.27E+05	3.90E-07	0.72732	0.99985	0.99932
4	2.03E+05	8.82E-08	2.17E+04	1.09E-07	6.66E+04	6.76E-07	0.70114	0.99986	0.9991
5	1.67E+04	2.46E-07	1.12E+05	2.23E-07	2.49E+05	4.59E-07	0.75584	0.99992	0.99568
6	1.75E+04	2.04E-07	1.21E+05	1.70E-07	3.22E+05	9.45E-07	0.68164	0.99995	0.99493

40	R0	C0	R1	C1	R2	Q	a	R^2	R^2 phi
1	1.49E+04	1.24E-07	8.64E+04	1.10E-07	5.80E+05	6.63E-07	0.69105	0.99999	0.99921
2	4.92E+03	2.98E-07	1.15E+04	4.75E-07	1.00E+05	4.31E-07	0.71755	0.9999	0.99974
3	1.16E+04	1.08E-07	3.63E+04	1.82E-07	9.19E+04	5.68E-07	0.69835	0.99987	0.99939
4	1.60E+05	9.27E-08	1.67E+04	1.23E-07	1.53E+05	5.70E-07	0.7162	0.9999	0.99924
5	1.31E+04	2.64E-07	1.21E+05	1.74E-07	3.19E+05	4.93E-07	0.74875	0.99999	0.9903
6	1.12E+04	2.51E-07	1.20E+05	1.36E-07	5.17E+05	9.82E-07	0.6789	0.99991	0.9942

50	R0	C0	R1	C1	R2	Q	a	R^2	R^2 phi
1	1.31E+04	1.35E-07	8.29E+04	1.03E-07	6.84E+05	6.58E-07	0.69073	0.99999	0.99827
2	6.98E+03	2.04E-07	2.36E+04	2.64E-07	1.04E+05	6.12E-07	0.68863	0.99998	0.99979
3	5.14E+03	1.40E-07	2.89E+04	1.02E-07	8.78E+04	6.82E-07	0.68285	0.99981	0.99959
4	8.72E+04	9.20E-08	8.09E+03	1.74E-07	1.00E+06	3.83E-07	0.74846	0.99979	0.99928
5	1.36E+04	2.40E-07	1.91E+05	1.23E-07	3.19E+05	8.48E-07	0.70384	0.99997	0.99614
6	1.60E+04	2.09E-07	1.26E+05	1.43E-07	5.31E+05	1.00E-06	0.67659	0.99999	0.99466

60	R0	C0	R1	C1	R2	Q	a	R^2	R^2 phi
1	1.41E+04	1.20E-07	9.38E+04	9.49E-08	5.08E+05	7.99E-07	0.67602	0.99999	0.99972
2	7.01E+03	1.97E-07	2.12E+04	2.48E-07	9.75E+04	6.31E-07	0.68613	0.99998	0.99985
3	1.08E+04	9.95E-08	3.41E+04	1.30E-07	8.94E+04	8.24E-07	0.66791	0.99996	0.99994
4	9.77E+04	9.98E-08	1.45E+04	1.39E-07	9.12E+05	3.97E-07	0.74282	0.99999	0.99896
5	1.38E+04	2.27E-07	2.20E+05	1.23E-07	3.53E+05	8.43E-07	0.70289	0.99993	0.99648
6	3.15E+04	1.78E-07	1.07E+05	2.64E-07	8.27E+05	6.27E-07	0.71594	0.99997	0.98774
