# Supplementary material for: Impedance Characterization and Modeling of Gold, Silver, and PEDOT:PSS Ultra-Thin Tattoo Electrodes for Wearable Bioelectronics
Source: Sensors (Basel). 2025 Jul 23;25(15):4568. doi: 10.3390/s25154568 (PMC12349162; doi:10.3390/s25154568)
Supplement: Supplementary file 1 [file sensors-25-04568-s001.zip › DATA Supplementary/Table S3. Data_evaluation_Ag.pdf]

Sub Model 0

0	R0	R1	C1	R^2	R^2 phi
1	1.42E+03	1.07E+05	4.71E-08	0.98808	0.14353
2	1.51E+03	1.24E+05	4.46E-08	0.99297	0.40983
3	1.42E+03	6.27E+04	5.17E-08	0.98877	0.7054
4	1.51E+03	1.16E+05	3.78E-08	0.98816	0.52483
5	1.07E+03	7.94E+04	6.77E-08	0.99037	0.12242
6	1.38E+03	9.59E+04	6.64E-08	0.9898	-1.24043

10	R0	R1	C1	R^2	R^2 phi
1	1.34E+03	1.42E+05	4.16E-08	0.99217	0.41989
2	1.48E+03	1.27E+05	4.29E-08	0.9934	0.48067
3	1.34E+03	8.42E+04	4.99E-08	0.99019	0.57025
4	1.51E+03	1.13E+05	3.66E-08	0.98937	0.53169
5	9.96E+02	1.17E+05	6.32E-08	0.99214	-0.29237
6	1.38E+03	1.10E+05	6.20E-08	0.99222	-1.28871

20	R0	R1	C1	R^2	R^2 phi
1	1.38E+03	1.67E+05	3.79E-08	0.99328	0.43117
2	1.46E+03	1.35E+05	4.13E-08	0.99398	0.53415
3	1.33E+03	9.49E+04	4.80E-08	0.99137	0.53629
4	1.50E+03	1.21E+05	3.55E-08	0.9904	0.56004
5	9.87E+02	1.20E+05	6.27E-08	0.99204	-0.28447
6	1.37E+03	1.31E+05	5.90E-08	0.99125	-2.00327

30	R0	R1	C1	R^2	R^2 phi
1	1.30E+03	1.95E+05	3.72E-08	0.99453	0.46937
2	1.47E+03	1.28E+05	4.07E-08	0.99408	0.62277
3	1.31E+03	1.02E+05	4.68E-08	0.99219	0.52079
4	1.49E+03	1.34E+05	3.51E-08	0.99068	0.54676
5	9.94E+02	1.27E+05	5.97E-08	0.99303	-0.20744
6	1.38E+03	1.36E+05	5.74E-08	0.99135	-2.17558

40	R0	R1	C1	R^2	R^2 phi
1	1.28E+03	2.28E+05	3.50E-08	0.99496	0.43711
2	1.43E+03	1.51E+05	4.03E-08	0.99438	0.53711
3	1.30E+03	1.13E+05	4.49E-08	0.99308	0.51747
4	1.51E+03	1.45E+05	3.29E-08	0.99318	0.54444
5	9.82E+02	1.39E+05	5.93E-08	0.99307	-0.24856
6	1.37E+03	1.46E+05	5.57E-08	0.99172	-2.29012

50	R0	R1	C1	R^2	R^2 phi
1	1.24E+03	2.40E+05	3.45E-08	0.99465	0.31646
2	1.47E+03	1.52E+05	3.91E-08	0.9944	0.54748
3	1.33E+03	1.13E+05	4.31E-08	0.99271	0.62325
4	1.50E+03	1.62E+05	3.40E-08	0.9916	0.46164
5	9.85E+02	1.47E+05	5.76E-08	0.99343	-0.20326
6	1.36E+03	1.48E+05	5.51E-08	0.99134	-2.24162

60	R0	R1	C1	R^2	R^2 phi
1	1.24E+03	2.70E+05	3.38E-08	0.99442	0.58654
2	1.46E+03	1.66E+05	3.91E-08	0.99415	0.45011
3	1.28E+03	1.26E+05	4.38E-08	0.99251	0.50986
4	1.49E+03	1.72E+05	3.33E-08	0.99298	0.4158
5	9.93E+02	1.58E+05	5.69E-08	0.99374	-0.35528
6	1.36E+03	1.54E+05	5.39E-08	0.99178	-2.18597

Model 1

0	R0	R1	C0	R2	Q	a	R3	R^2	R^2 phi
1	1.00E+01	1.60E+05	1.74E-07	7.55E+04	1.59E-07	0.80538	10.01269	0.99995	0.99821
2	1.29E+02	7.60E+03	2.47E-07	2.23E+05	1.63E-07	0.80208	31.89508	0.99999	0.99964
3	1.25E+01	5.52E+03	2.26E-07	9.12E+04	2.49E-07	0.77223	12.54771	0.99987	0.9996
4	1.00E+01	1.83E+04	8.11E-08	2.13E+05	1.87E-07	0.79041	10.00002	0.99998	0.99936
5	1.00E+01	1.00E+05	3.09E-07	7.03E+04	2.62E-07	0.78679	10	0.99998	0.99949
6	1.06E+02	9.97E+05	6.56E-07	1.51E+05	2.83E-07	0.76093	105.97657	0.99998	0.9986

10	R0	R1	C0	R2	Q	a	R3	R^2	R^2 phi
1	1.00E+01	1.74E+05	1.38E-07	9.44E+04	1.37E-07	0.82307	10.00224	0.99995	0.99843
2	1.07E+02	1.10E+04	1.94E-07	2.31E+05	1.62E-07	0.804	27.16559	0.99999	0.99954
3	1.07E+01	7.81E+03	1.86E-07	1.39E+05	2.23E-07	0.78341	10.69266	0.99999	0.99976
4	1.00E+01	1.41E+05	1.49E-07	7.60E+04	9.28E-08	0.84707	10	0.99995	0.99973
5	1.00E+01	2.02E+05	2.00E-07	8.76E+04	2.53E-07	0.79443	10.02832	0.99995	0.9973
6	1.07E+02	9.90E+05	6.66E-07	1.84E+05	2.46E-07	0.77328	96.33516	0.99989	0.99676

20	R0	R1	C0	R2	Q	a	R3	R^2	R^2 phi
1	1.96E+02	2.13E+05	7.23E-08	5.08E+04	1.15E-07	0.86399	196.08878	0.99965	0.99918
2	9.58E+01	1.35E+04	1.78E-07	2.42E+05	1.56E-07	0.80801	22.72424	0.99998	0.99943
3	1.17E+01	8.28E+04	2.72E-07	8.65E+04	1.59E-07	0.81145	11.68678	0.99996	0.99849
4	1.00E+01	1.53E+05	1.57E-07	8.83E+04	9.28E-08	0.84494	10	0.99993	0.9981
5	1.00E+01	2.33E+05	1.94E-07	8.85E+04	2.48E-07	0.797	10.03596	0.99993	0.99714
6	1.39E+02	2.10E+05	1.25E-07	6.00E+04	3.22E-07	0.76101	137.37423	0.99996	0.99563

30	R0	R1	C0	R2	Q	a	R3	R^2	R^2 phi
1	1.71E+01	2.60E+05	1.05E-07	1.19E+05	1.23E-07	0.83595	17.08176	0.99996	0.9989
2	7.73E+01	1.43E+04	1.68E-07	2.15E+05	1.58E-07	0.80702	19.00787	0.99997	0.99934
3	1.04E+01	1.14E+05	2.63E-07	9.20E+04	1.46E-07	0.81925	10.40501	0.99999	0.99873
4	1.04E+01	2.18E+05	1.82E-07	1.12E+05	9.35E-08	0.84175	10.35993	0.99954	0.99315
5	1.04E+01	1.31E+04	2.43E-07	3.95E+05	2.35E-07	0.79746	10.37353	0.99997	0.99687
6	1.28E+02	2.43E+05	1.32E-07	7.33E+04	3.01E-07	0.76384	126.77757	0.99999	0.99908

40	R0	R1	C0	R2	Q	a	R3	R^2	R^2 phi
1	1.08E+01	3.47E+05	7.70E-08	9.98E+04	1.16E-07	0.85182	171.23051	0.99994	0.99933
2	1.00E+01	2.35E+04	1.46E-07	2.95E+05	1.74E-07	0.79622	10	0.99997	0.99619
3	1.15E+01	2.53E+05	3.12E-07	1.16E+05	1.30E-07	0.8277	22.0259	0.99992	0.99619
4	6.85E+01	3.61E+05	1.90E-07	1.29E+05	8.17E-08	0.85273	68.54141	0.9998	0.9928
5	1.00E+01	1.49E+04	2.61E-07	4.54E+05	2.27E-07	0.8009	10.01113	0.99998	0.99271
6	9.24E+01	2.51E+05	1.14E-07	6.55E+04	3.19E-07	0.75709	85.42478	0.99999	0.99712

50	R0	R1	C0	R2	Q	a	R3	R^2	R^2 phi
1	2.00E+01	9.84E+05	1.19E-07	1.79E+05	9.57E-08	0.85335	10.00236	0.99996	0.99272
2	3.47E+02	1.76E+05	8.10E-08	5.33E+04	1.10E-07	0.87525	346.64648	0.99993	0.99595
3	1.99E+02	1.16E+05	1.12E-07	5.24E+04	1.18E-07	0.86059	175.09794	0.99997	0.99789
4	1.01E+01	1.70E+04	9.20E-08	3.04E+05	1.21E-07	0.82556	10.14402	0.99904	0.99876
5	2.56E+01	1.67E+04	2.39E-07	4.92E+05	2.14E-07	0.80744	25.566	0.99997	0.99509
6	4.73E+01	2.91E+05	1.39E-07	9.53E+04	2.94E-07	0.75804	44.36264	0.99998	0.99524

60	R0	R1	C0	R2	Q	a	R3	R^2	R^2 phi
1	2.59E+01	3.58E+05	6.45E-08	9.94E+04	1.32E-07	0.84829	191.17202	0.99947	0.95944
2	3.90E+02	2.05E+05	6.83E-08	4.17E+04	1.09E-07	0.88593	389.89367	0.99996	0.99429
3	1.00E+01	1.38E+05	1.34E-07	7.70E+04	1.48E-07	0.82396	10.00705	0.99972	0.99954
4	1.03E+01	2.51E+05	1.04E-07	1.03E+05	9.53E-08	0.84335	10.32471	0.99958	0.99457
5	6.74E+01	1.24E+04	2.92E-07	4.79E+05	1.82E-07	0.8233	44.07961	0.99996	0.99383
6	8.40E+01	2.73E+05	1.07E-07	6.47E+04	3.02E-07	0.76135	77.79576	0.99999	0.9944

Model 2

0	R0	R1	C0	R2	Q	a	R^2	R^2 phi
1	1.00E+01	1.58E+05	1.69E-07	7.34E+04	1.59E-07	0.80554	0.99995	0.99845
2	1.89E+02	1.16E+05	1.64E-07	9.21E+04	1.63E-07	0.80688	0.99999	0.99948
3	1.14E+01	5.54E+03	2.29E-07	9.14E+04	2.51E-07	0.77082	0.99987	0.9996
4	1.00E+01	1.83E+04	8.14E-08	2.13E+05	1.88E-07	0.78939	0.99998	0.99937
5	1.29E+01	1.00E+05	3.12E-07	7.10E+04	2.62E-07	0.7861	0.99998	0.99947
6	2.15E+02	4.52E+05	6.11E-07	1.47E+05	2.85E-07	0.76076	0.99998	0.99861

10	R0	R1	C0	R2	Q	a	R^2	R^2 phi
1	1.00E+01	1.73E+05	1.35E-07	9.29E+04	1.38E-07	0.82245	0.99995	0.99854
2	2.20E+02	1.26E+05	1.56E-07	9.16E+04	1.44E-07	0.82074	0.99999	0.99953
3	1.03E+01	7.75E+03	1.88E-07	1.40E+05	2.24E-07	0.78257	0.99999	0.99977
4	1.28E+01	1.41E+05	1.50E-07	7.60E+04	9.30E-08	0.84665	0.99995	0.99973
5	1.11E+01	2.05E+05	1.90E-07	8.30E+04	2.59E-07	0.79267	0.99995	0.99766
6	2.15E+02	7.00E+05	5.70E-07	1.73E+05	2.48E-07	0.77315	0.99989	0.99725

20	R0	R1	C0	R2	Q	a	R^2	R^2 phi
1	4.16E+02	2.13E+05	7.20E-08	5.02E+04	1.13E-07	0.86669	0.99966	0.9991
2	2.44E+02	1.34E+05	1.54E-07	9.79E+04	1.31E-07	0.83031	0.99999	0.99955
3	1.00E+01	8.49E+04	2.33E-07	7.92E+04	1.62E-07	0.81104	0.99997	0.99871
4	1.56E+01	1.54E+05	1.59E-07	8.89E+04	9.34E-08	0.84396	0.99993	0.99808
5	1.00E+01	2.33E+05	1.97E-07	8.97E+04	2.49E-07	0.79608	0.99993	0.99704
6	3.28E+02	2.11E+05	1.18E-07	5.36E+04	3.12E-07	0.7673	0.99996	0.99651

30	R0	R1	C0	R2	Q	a	R^2	R^2 phi
1	4.57E+01	2.60E+05	1.03E-07	1.16E+05	1.23E-07	0.83646	0.99996	0.99885
2	2.25E+02	1.12E+05	1.76E-07	1.00E+05	1.23E-07	0.8347	0.99999	0.99943
3	1.00E+01	1.15E+05	2.69E-07	9.31E+04	1.46E-07	0.81841	0.99999	0.99875
4	1.08E+01	2.16E+05	1.80E-07	1.11E+05	9.37E-08	0.84151	0.99954	0.99329
5	2.12E+02	2.43E+05	1.49E-07	6.87E+04	2.13E-07	0.82302	0.99997	0.99763
6	2.58E+02	2.43E+05	1.31E-07	7.27E+04	3.01E-07	0.76411	0.99999	0.99909

40	R0	R1	C0	R2	Q	a	R^2	R^2 phi
1	2.09E+02	3.47E+05	7.52E-08	9.56E+04	1.16E-07	0.8538	0.99994	0.99922
2	2.31E+02	1.78E+05	1.98E-07	1.33E+05	1.16E-07	0.8386	0.99999	0.99731
3	4.01E+01	2.65E+05	3.12E-07	1.16E+05	1.30E-07	0.82822	0.99992	0.99619
4	1.03E+01	2.54E+05	1.57E-07	1.15E+05	8.37E-08	0.8503	0.99974	0.99642
5	1.53E+02	3.08E+05	1.64E-07	8.93E+04	2.16E-07	0.81777	0.99999	0.99431
6	1.77E+02	2.51E+05	1.12E-07	6.32E+04	3.20E-07	0.75701	0.99999	0.99725

50	R0	R1	C0	R2	Q	a	R^2	R^2 phi
1	1.86E+02	5.09E+05	8.33E-08	1.18E+05	1.01E-07	0.86147	0.99994	0.99736
2	5.84E+02	1.74E+05	8.44E-08	5.81E+04	1.17E-07	0.86264	0.99994	0.99573
3	3.34E+02	1.15E+05	1.15E-07	5.41E+04	1.21E-07	0.85608	0.99998	0.99784
4	1.71E+01	1.67E+04	9.27E-08	3.02E+05	1.20E-07	0.82632	0.99904	0.99871
5	2.25E+02	3.30E+05	1.49E-07	8.64E+04	2.02E-07	0.82821	0.99996	0.99635
6	9.24E+01	2.91E+05	1.38E-07	9.51E+04	2.94E-07	0.75808	0.99998	0.99525

60	R0	R1	C0	R2	Q	a	R^2	R^2 phi
1	2.07E+02	3.59E+05	6.29E-08	9.49E+04	1.36E-07	0.84575	0.99947	0.95937
2	7.82E+02	2.05E+05	6.82E-08	4.17E+04	1.09E-07	0.88619	0.99996	0.99429
3	1.79E+01	1.39E+05	1.33E-07	7.63E+04	1.49E-07	0.8238	0.99972	0.99953
4	1.03E+01	2.69E+05	1.25E-07	1.22E+05	9.18E-08	0.84538	0.99964	0.99021
5	2.12E+02	3.30E+05	1.32E-07	8.26E+04	2.20E-07	0.82154	0.99995	0.99507
6	1.69E+02	2.77E+05	1.06E-07	6.29E+04	2.95E-07	0.76451	0.99998	0.99564

Model 3

0	R0	C0	R1	C1	R2	Q	a	R^2	R^2 phi
1	2.94E+05	4.24E-07	7.24E+03	1.78E-07	1.37E+05	2.14E-07	0.78001	0.99997	0.99937
2	3.95E+04	3.74E-07	7.31E+04	3.83E-07	1.07E+05	1.75E-07	0.79277	0.99999	0.99887
3	1.45E+04	2.64E-07	5.89E+03	1.69E-07	8.31E+04	4.55E-07	0.72394	0.99993	0.99963
4	3.49E+05	6.81E-07	8.99E+04	1.32E-07	6.16E+04	1.05E-07	0.84042	0.99999	0.99986
5	4.85E+03	2.76E-07	1.52E+04	3.34E-07	2.07E+05	4.89E-07	0.73351	0.99999	0.9997
6	2.47E+04	4.39E-07	6.82E+05	5.31E-07	1.05E+05	4.20E-07	0.72035	0.99997	0.99577

10	R0	C0	R1	C1	R2	Q	a	R^2	R^2 phi
1	2.56E+05	4.17E-07	5.86E+03	2.26E-07	1.80E+05	1.40E-07	0.81957	0.99999	0.99945
2	1.27E+04	1.38E-07	5.23E+04	1.46E-07	2.51E+05	4.51E-07	0.71795	0.99999	0.99973
3	1.65E+04	3.24E-07	8.64E+03	1.53E-07	1.34E+05	3.60E-07	0.74535	1	0.9998
4	1.48E+05	1.52E-07	2.41E+04	1.73E-07	5.06E+04	1.16E-07	0.83544	0.99996	0.99957
5	5.60E+03	2.55E-07	3.91E+04	2.06E-07	7.35E+05	4.87E-07	0.74016	0.99999	0.99928
6	3.08E+04	2.96E-07	5.23E+05	3.67E-07	9.95E+04	4.56E-07	0.71436	0.99992	0.99305

20	R0	C0	R1	C1	R2	Q	a	R^2	R^2 phi
1	1.88E+05	8.21E-08	1.81E+04	1.08E-07	6.14E+04	4.67E-07	0.73036	0.99963	0.99879
2	1.36E+04	1.34E-07	5.70E+04	1.32E-07	2.77E+05	4.46E-07	0.72078	0.99999	0.99971
3	2.19E+04	2.21E-07	7.86E+03	1.60E-07	1.72E+05	3.57E-07	0.74774	1	0.99915
4	4.00E+05	3.45E-07	9.31E+03	1.23E-07	1.31E+05	1.27E-07	0.82255	1	0.99976
5	5.97E+03	2.55E-07	4.04E+04	2.05E-07	7.57E+05	4.72E-07	0.74469	0.99998	0.99756
6	9.26E+04	2.68E-07	1.04E+05	2.91E-07	7.93E+04	3.76E-07	0.7337	0.99996	0.98811

30	R0	C0	R1	C1	R2	Q	a	R^2	R^2 phi
1	2.86E+05	7.86E-08	5.26E+04	7.87E-08	1.72E+04	3.58E-07	0.77018	0.99997	0.99925
2	1.27E+04	1.31E-07	5.54E+04	1.20E-07	2.41E+05	4.76E-07	0.7166	0.99999	0.99982
3	3.10E+04	1.37E-07	6.01E+03	1.69E-07	2.32E+05	4.13E-07	0.73818	1	0.99952
4	9.78E+05	2.99E-07	6.10E+03	1.57E-07	1.41E+05	1.09E-07	0.83414	0.99978	0.99712
5	1.15E+04	2.04E-07	4.85E+04	2.32E-07	7.80E+05	4.79E-07	0.74142	0.99995	0.99858
6	1.02E+05	3.04E-07	1.44E+05	3.76E-07	1.13E+05	3.24E-07	0.74358	0.99998	0.99592

40	R0	C0	R1	C1	R2	Q	a	R^2	R^2 phi
1	3.60E+05	6.44E-08	4.97E+04	7.63E-08	1.51E+04	4.58E-07	0.75348	0.99997	0.99904
2	1.03E+04	2.06E-07	3.76E+04	1.58E-07	3.21E+05	2.58E-07	0.76595	0.99999	0.99661
3	4.22E+04	1.01E-07	4.29E+03	1.95E-07	3.74E+05	4.44E-07	0.73417	0.99992	0.9971
4	2.35E+05	1.47E-07	1.81E+04	1.60E-07	9.38E+04	1.29E-07	0.81958	0.99974	0.99555
5	6.55E+03	3.25E-07	3.74E+04	2.22E-07	9.01E+05	3.63E-07	0.76417	0.99999	0.99389
6	1.38E+05	2.14E-07	1.06E+05	2.93E-07	7.98E+04	3.48E-07	0.74032	0.99999	0.9941

50	R0	C0	R1	C1	R2	Q	a	R^2	R^2 phi
1	4.92E+05	7.31E-08	5.32E+04	9.08E-08	3.74E+04	3.16E-07	0.77492	0.99995	0.99653
2	2.38E+04	9.43E-08	1.61E+05	8.53E-08	5.13E+04	8.63E-07	0.6695	0.99993	0.99644
3	5.92E+04	1.53E-07	1.71E+04	1.01E-07	1.31E+05	5.26E-07	0.71863	0.99998	0.99759
4	8.32E+04	8.72E-08	1.82E+04	6.98E-08	7.89E+05	4.66E-07	0.72396	0.99944	0.99723
5	1.92E+04	1.76E-07	9.37E+04	2.02E-07	9.05E+05	5.80E-07	0.7265	0.99997	0.9924
6	5.18E+05	2.03E-07	6.75E+04	1.87E-07	5.62E+04	3.90E-07	0.73333	0.99999	0.99478

60	R0	C0	R1	C1	R2	Q	a	R^2	R^2 phi
1	3.86E+05	5.43E-08	4.76E+04	8.21E-08	1.12E+04	5.08E-07	0.75137	0.99953	0.95945
2	1.92E+04	1.01E-07	1.82E+05	7.62E-08	5.67E+04	9.00E-07	0.66691	0.99995	0.99439
3	6.30E+04	1.21E-07	1.30E+04	1.11E-07	3.18E+05	5.62E-07	0.71611	0.99988	0.99903
4	6.21E+05	2.68E-07	1.11E+04	1.24E-07	1.98E+05	1.13E-07	0.8298	0.99978	0.99448
5	1.68E+04	1.96E-07	1.07E+05	1.82E-07	8.59E+05	5.47E-07	0.73162	0.99997	0.99122
6	1.49E+05	2.01E-07	1.21E+05	2.62E-07	7.60E+04	3.29E-07	0.74604	0.99998	0.99171
