# Supplementary material for: Impedance Characterization and Modeling of Gold, Silver, and PEDOT:PSS Ultra-Thin Tattoo Electrodes for Wearable Bioelectronics
Source: Sensors (Basel). 2025 Jul 23;25(15):4568. doi: 10.3390/s25154568 (PMC12349162; doi:10.3390/s25154568)
Supplement: Supplementary file 1 [file sensors-25-04568-s001.zip › DATA Supplementary/Table S4. Data_evaluation_AgAgCl.pdf]

Sub Model 0

0	R0	R1	C1	R^2	R^2 phi
1	1.25E+03	3.32E+05	3.57E-08	0.99468	-0.52857
2	2.06E+03	2.27E+05	2.81E-08	0.99723	0.71325
3	1.49E+03	9.97E+04	4.04E-08	0.99528	0.82322
4	1.55E+03	2.02E+05	3.77E-08	0.99203	-0.36318
5	1.20E+03	1.61E+05	5.12E-08	0.99542	0.26812
6	1.59E+03	1.30E+05	3.93E-08	0.996	0.76014

10	R0	R1	C1	R^2	R^2 phi
1	1.22E+03	3.13E+05	3.71E-08	0.99427	-0.52302
2	2.11E+03	1.42E+05	2.82E-08	0.99592	0.9019
3	1.52E+03	6.02E+04	4.59E-08	0.98936	0.8383
4	1.55E+03	2.04E+05	3.76E-08	0.99306	0.11019
5	1.21E+03	1.57E+05	5.15E-08	0.99534	0.1435
6	1.56E+03	1.16E+05	4.04E-08	0.99462	0.79428

20	R0	R1	C1	R^2	R^2 phi
1	1.21E+03	2.95E+05	3.77E-08	0.9943	-0.49216
2	2.09E+03	1.68E+05	2.79E-08	0.99772	0.89429
3	1.48E+03	6.91E+04	4.31E-08	0.995	0.86026
4	1.58E+03	2.05E+05	3.69E-08	0.99162	-0.0914
5	1.24E+03	1.54E+05	5.08E-08	0.99529	0.23312
6	1.53E+03	1.69E+05	3.88E-08	0.99587	0.6739

30	R0	R1	C1	R^2	R^2 phi
1	1.27E+03	3.06E+05	3.68E-08	0.99196	-0.13532
2	2.12E+03	1.47E+05	2.78E-08	0.99744	0.91612
3	1.47E+03	7.29E+04	4.31E-08	0.99479	0.8561
4	1.54E+03	1.99E+05	3.79E-08	0.99242	-0.0629
5	1.24E+03	1.55E+05	5.03E-08	0.99481	0.38537
6	1.57E+03	1.81E+05	3.83E-08	0.99645	0.68357

40	R0	R1	C1	R^2	R^2 phi
1	1.26E+03	2.76E+05	3.77E-08	0.99322	-0.23288
2	2.15E+03	1.24E+05	3.13E-08	0.99463	0.80111
3	1.43E+03	7.71E+04	4.38E-08	0.99335	0.84414
4	2.83E+03	1.94E+05	3.68E-08	0.99181	-1.10709
5	1.25E+03	1.49E+05	5.02E-08	0.99528	0.36802
6	1.59E+03	1.83E+05	3.79E-08	0.99655	0.60159

50	R0	R1	C1	R^2	R^2 phi
1	1.25E+03	2.73E+05	3.82E-08	0.99442	-0.07859
2	2.19E+03	1.61E+05	2.88E-08	0.9962	0.87011
3	1.51E+03	5.75E+04	4.43E-08	0.99234	0.87882
4	1.55E+03	1.99E+05	3.75E-08	0.99241	0.22941
5	1.24E+03	1.55E+05	5.10E-08	0.99521	0.37008
6	1.54E+03	1.65E+05	3.88E-08	0.9963	0.72313

60	R0	R1	C1	R^2	R^2 phi
1	1.26E+03	2.70E+05	3.86E-08	0.99457	-0.05177
2	2.29E+03	1.05E+05	3.02E-08	0.99644	0.86699
3	1.46E+03	7.83E+04	4.13E-08	0.99645	0.8718
4	1.55E+03	1.99E+05	3.72E-08	0.99265	0.22864
5	1.25E+03	1.41E+05	5.17E-08	0.99528	0.40776
6	1.56E+03	1.78E+05	3.85E-08	0.99609	0.64271

Model 1

0	R0	R1	C0	R2	Q	a	R3	R^2	R^2 phi
1	2.23E+02	8.23E+05	5.88E-08	8.69E+04	1.40E-07	0.85369	226.05299	0.99998	0.99772
2	8.43E+02	2.05E+04	7.10E-08	2.88E+05	4.88E-08	0.94503	844.18138	0.99993	0.99625
3	7.08E+01	2.01E+02	7.28E-06	1.35E+05	1.09E-07	0.83589	10.37396	0.99948	0.99306
4	1.08E+01	3.82E+05	8.87E-08	9.95E+04	1.50E-07	0.80412	10.75623	0.99994	0.95678
5	1.78E+02	9.66E+05	4.72E-07	2.06E+05	1.33E-07	0.84632	161.17203	0.99979	0.99602
6	2.64E+02	3.45E+04	9.56E-08	1.95E+05	1.98E-07	0.80499	263.7681	0.99993	0.99841

10	R0	R1	C0	R2	Q	a	R3	R^2	R^2 phi
1	2.95E+02	8.38E+05	6.52E-08	9.89E+04	1.47E-07	0.84389	38.51279	0.99999	0.99366
2	5.75E+02	2.74E+04	7.37E-08	1.59E+05	8.51E-08	0.87845	664.24766	0.99992	0.99966
3	1.00E+01	2.79E+04	2.85E-07	4.93E+04	1.37E-07	0.83153	258.42206	0.99976	0.99971
4	1.40E+01	2.74E+05	8.86E-08	1.09E+05	1.71E-07	0.79554	17.53765	0.99993	0.9846
5	4.58E+02	9.64E+03	1.42E-07	2.54E+05	1.01E-07	0.90958	412.56905	0.99994	0.98876
6	2.49E+02	2.18E+04	1.26E-07	1.59E+05	1.63E-07	0.82078	248.56153	0.99943	0.99929

20	R0	R1	C0	R2	Q	a	R3	R^2	R^2 phi
1	3.15E+02	8.43E+05	6.85E-08	9.85E+04	1.38E-07	0.85087	62.57391	0.99985	0.98619
2	4.45E+02	5.34E+04	6.49E-08	1.88E+05	1.25E-07	0.83544	498.54876	0.99999	0.99976
3	3.97E+02	9.17E+05	1.32E-06	7.93E+04	1.04E-07	0.85614	10.18452	0.99977	0.99842
4	2.77E+02	3.58E+05	8.14E-08	8.94E+04	1.29E-07	0.8315	114.34495	0.99944	0.99093
5	4.35E+02	9.39E+03	1.62E-07	2.54E+05	1.07E-07	0.89741	391.12758	0.9999	0.99396
6	2.33E+02	5.92E+04	7.86E-08	4.06E+05	2.46E-07	0.78612	237.60457	0.99996	0.99522

30	R0	R1	C0	R2	Q	a	R3	R^2	R^2 phi
1	6.15E+02	6.15E+05	5.56E-08	6.04E+04	1.29E-07	0.8813	113.13307	0.99842	0.93374
2	4.46E+02	5.38E+04	5.70E-08	1.52E+05	1.51E-07	0.82196	498.89557	0.99997	0.99975
3	1.03E+01	5.35E+04	1.33E-06	8.39E+04	1.05E-07	0.85323	373.04898	0.99979	0.99849
4	1.00E+01	3.33E+05	1.12E-07	1.38E+05	1.48E-07	0.8041	10	0.9994	0.99747
5	2.51E+02	1.14E+04	2.78E-07	2.86E+05	1.37E-07	0.85026	235.13353	0.99966	0.98924
6	3.15E+02	2.08E+04	1.87E-07	2.83E+05	1.08E-07	0.85498	321.5681	0.99995	0.99208

40	R0	R1	C0	R2	Q	a	R3	R^2	R^2 phi
1	4.81E+02	6.71E+05	6.64E-08	8.19E+04	1.24E-07	0.86834	90.18132	0.99994	0.9903
2	1.00E+01	5.75E+04	5.32E-08	2.37E+05	5.01E-07	0.67699	10	0.99947	0.99344
3	5.39E+01	5.97E+05	2.00E-06	9.65E+04	1.23E-07	0.8298	10.24493	0.99989	0.99835
4	9.83E+02	6.03E+05	1.59E-07	1.73E+05	1.14E-07	0.82638	983.41006	0.99993	0.96295
5	2.66E+02	1.27E+04	2.28E-07	2.80E+05	1.42E-07	0.84872	249.24667	0.99987	0.99819
6	5.29E+02	1.47E+04	1.25E-07	2.70E+05	8.02E-08	0.90316	540.4575	0.99994	0.99467

50	R0	R1	C0	R2	Q	a	R3	R^2	R^2 phi
1	3.12E+02	5.40E+05	6.83E-08	8.82E+04	1.52E-07	0.84273	63.89259	0.99997	0.99114
2	3.78E+02	3.70E+04	8.88E-08	1.89E+05	1.10E-07	0.83597	547.84264	0.99954	0.99636
3	1.00E+01	1.62E+04	1.17E-06	6.34E+04	1.17E-07	0.84176	279.58997	0.99975	0.99925
4	1.24E+01	2.46E+05	7.35E-08	7.73E+04	1.88E-07	0.79091	12.37933	0.99932	0.99609
5	4.82E+02	8.12E+03	1.44E-07	2.26E+05	8.99E-08	0.92519	453.86606	0.99985	0.99673
6	1.93E+02	7.50E+04	7.10E-08	4.93E+05	3.82E-07	0.73794	10.05421	0.99989	0.993

60	R0	R1	C0	R2	Q	a	R3	R^2	R^2 phi
1	3.86E+02	5.05E+05	6.34E-08	7.17E+04	1.71E-07	0.83288	73.19104	0.99995	0.97014
2	7.28E+02	9.96E+05	5.99E-07	1.09E+05	5.47E-08	0.89906	727.70718	0.99973	0.99487
3	1.00E+01	9.56E+05	9.75E-07	8.66E+04	8.95E-08	0.87165	475.98162	0.99977	0.99896
4	1.00E+01	2.51E+05	8.85E-08	1.05E+05	1.62E-07	0.79983	10	0.99965	0.99559
5	8.32E+01	3.33E+04	1.74E-07	2.98E+05	2.52E-07	0.7842	78.32905	0.99975	0.99445
6	5.43E+02	1.29E+04	1.24E-07	2.51E+05	7.60E-08	0.91323	556.12607	0.99983	0.99737

Model 2

0	R0	R1	C0	R2	Q	a	R^2	R^2 phi
1	3.76E+02	8.55E+05	6.10E-08	9.73E+04	1.43E-07	0.84768	0.99999	0.99753
2	1.48E+03	2.50E+05	4.49E-08	4.74E+04	8.42E-08	0.90724	0.99993	0.99753
3	1.49E+02	1.48E+04	7.01E-07	1.17E+05	1.11E-07	0.83828	0.99945	0.99375
4	3.44E+02	3.75E+05	7.80E-08	7.69E+04	1.29E-07	0.83093	0.99993	0.96399
5	3.28E+02	9.95E+05	4.71E-07	2.08E+05	1.34E-07	0.84457	0.99979	0.99579
6	5.91E+02	3.13E+04	9.89E-08	1.91E+05	1.79E-07	0.81635	0.99993	0.99877

10	R0	R1	C0	R2	Q	a	R^2	R^2 phi
1	3.49E+02	8.47E+05	6.54E-08	9.93E+04	1.45E-07	0.84565	0.99999	0.99355
2	1.37E+03	9.16E+04	1.05E-07	8.62E+04	5.57E-08	0.91443	0.99995	0.99975
3	2.11E+02	2.49E+04	3.34E-07	5.30E+04	1.41E-07	0.82582	0.99976	0.99961
4	1.91E+02	2.80E+05	7.75E-08	8.49E+04	1.69E-07	0.80531	0.99994	0.98354
5	7.83E+02	2.08E+05	7.72E-08	2.83E+04	1.70E-07	0.87894	0.99995	0.9921
6	4.81E+02	2.24E+04	1.24E-07	1.60E+05	1.67E-07	0.81812	0.99943	0.99927

20	R0	R1	C0	R2	Q	a	R^2	R^2 phi
1	3.29E+02	8.46E+05	6.88E-08	1.01E+05	1.41E-07	0.84629	0.99985	0.98535
2	1.21E+03	8.23E+04	2.26E-07	1.52E+05	5.25E-08	0.90513	1	0.9999
3	3.19E+02	3.63E+04	9.91E-07	7.68E+04	1.07E-07	0.85176	0.99974	0.99895
4	3.85E+02	3.57E+05	8.16E-08	8.98E+04	1.29E-07	0.83092	0.99944	0.99101
5	7.83E+02	2.02E+05	8.07E-08	3.29E+04	1.72E-07	0.87308	0.99995	0.99613
6	4.72E+02	5.90E+04	7.87E-08	4.04E+05	2.45E-07	0.78658	0.99996	0.99521

30	R0	R1	C0	R2	Q	a	R^2	R^2 phi
1	7.30E+02	6.15E+05	5.56E-08	6.03E+04	1.28E-07	0.88165	0.99842	0.93366
2	1.21E+03	6.61E+04	3.58E-07	1.43E+05	5.00E-08	0.90638	0.99999	0.99985
3	2.93E+02	2.61E+04	9.34E-07	8.09E+04	1.09E-07	0.8481	0.99978	0.99925
4	1.00E+01	3.34E+05	1.14E-07	1.40E+05	1.48E-07	0.80366	0.9994	0.99746
5	5.98E+02	1.93E+05	1.09E-07	6.75E+04	1.78E-07	0.84666	0.99966	0.9887
6	6.33E+02	2.12E+04	1.85E-07	2.83E+05	1.09E-07	0.85436	0.99995	0.99209

40	R0	R1	C0	R2	Q	a	R^2	R^2 phi
1	5.56E+02	6.78E+05	6.70E-08	8.39E+04	1.24E-07	0.86687	0.99994	0.99084
2	9.18E+02	7.33E+05	4.21E-07	1.30E+05	6.87E-08	0.86748	0.99974	0.99433
3	1.49E+02	2.19E+04	9.97E-07	8.93E+04	1.20E-07	0.83655	0.99988	0.99833
4	1.95E+03	6.30E+05	1.57E-07	1.73E+05	1.14E-07	0.82576	0.99993	0.96341
5	6.27E+02	1.89E+05	1.13E-07	6.69E+04	1.66E-07	0.85284	0.99987	0.99837
6	1.06E+03	1.46E+04	1.27E-07	2.71E+05	8.08E-08	0.90147	0.99994	0.99472

50	R0	R1	C0	R2	Q	a	R^2	R^2 phi
1	3.89E+02	5.45E+05	6.87E-08	8.94E+04	1.51E-07	0.84308	0.99998	0.99084
2	1.19E+03	7.58E+04	1.77E-07	1.36E+05	6.26E-08	0.88724	0.99951	0.99661
3	2.93E+02	1.66E+04	9.98E-07	6.21E+04	1.16E-07	0.84242	0.99974	0.99922
4	2.04E+01	2.46E+05	7.32E-08	7.66E+04	1.89E-07	0.79054	0.99932	0.99608
5	7.41E+02	1.91E+05	7.35E-08	2.54E+04	2.04E-07	0.86293	0.99985	0.99849
6	1.77E+02	7.66E+04	7.01E-08	5.34E+05	4.01E-07	0.73248	0.99989	0.99303

60	R0	R1	C0	R2	Q	a	R^2	R^2 phi
1	3.26E+02	4.76E+05	6.50E-08	7.76E+04	1.72E-07	0.83155	0.99993	0.9854
2	1.10E+03	9.88E+05	6.80E-07	1.14E+05	6.30E-08	0.8764	0.99966	0.99775
3	4.24E+02	1.83E+05	9.99E-07	8.69E+04	9.22E-08	0.86669	0.99975	0.99936
4	1.01E+01	2.49E+05	9.24E-08	1.11E+05	1.60E-07	0.80001	0.99965	0.99572
5	4.48E+02	1.53E+05	2.59E-07	1.34E+05	1.46E-07	0.84352	0.99975	0.99607
6	1.06E+03	1.24E+04	1.32E-07	2.54E+05	7.76E-08	0.90796	0.99983	0.9976

Model 3

0	R0	C0	R1	C1	R2	Q	a	R^2	R^2 phi
1	3.33E+04	1.21E-07	6.53E+05	7.42E-08	3.02E+05	4.66E-07	0.73035	0.99999	0.99121
2	2.50E+04	9.31E-08	2.19E+05	5.06E-08	7.56E+04	8.76E-07	0.64008	0.9999	0.99442
3	1.31E+01	8.40E-07	1.00E+02	1.18E-07	1.33E+05	1.06E-07	0.83857	0.99944	0.99324
4	1.84E+05	1.67E-07	1.92E+05	1.68E-07	9.10E+04	1.51E-07	0.8045	0.99993	0.95872
5	7.13E+04	1.25E-07	3.18E+05	2.22E-07	6.05E+04	5.03E-07	0.72954	0.99979	0.99214
6	4.18E+04	7.67E-08	8.20E+04	1.61E-07	1.01E+05	9.88E-07	0.64688	0.99993	0.99812

10	R0	C0	R1	C1	R2	Q	a	R^2	R^2 phi
1	2.10E+04	1.91E-07	7.99E+05	7.06E-08	1.24E+05	3.27E-07	0.76363	0.99998	0.98971
2	5.50E+04	4.03E-08	1.14E+05	7.34E-08	5.91E+03	8.56E-07	0.64228	0.99996	0.99947
3	3.13E+02	3.12E-09	2.99E+04	2.59E-07	4.69E+04	1.34E-07	0.83614	0.99976	0.99975
4	1.31E+05	1.48E-07	1.54E+05	2.01E-07	1.04E+05	1.76E-07	0.7931	0.99993	0.98449
5	9.95E+03	2.25E-07	1.99E+05	8.33E-08	3.42E+04	9.02E-07	0.68162	0.99993	0.98533
6	1.38E+04	1.32E-07	6.32E+04	9.16E-08	2.59E+05	9.95E-07	0.64798	0.99963	0.99887

20	R0	C0	R1	C1	R2	Q	a	R^2	R^2 phi
1	2.08E+04	1.83E-07	8.03E+05	7.54E-08	1.29E+05	3.10E-07	0.76796	0.99982	0.98189
2	4.20E+04	6.85E-08	9.21E+04	7.54E-08	1.88E+05	1.00E-06	0.6239	0.99997	0.99899
3	3.18E+02	1.09E-09	3.69E+04	1.00E-06	7.69E+04	1.07E-07	0.8516	0.99974	0.99895
4	2.83E+04	9.90E-08	2.37E+05	1.07E-07	2.24E+05	4.67E-07	0.70713	0.99955	0.98535
5	1.43E+04	1.93E-07	1.99E+05	8.28E-08	2.54E+04	9.69E-07	0.67478	0.99994	0.99059
6	6.70E+04	6.87E-08	9.18E+04	2.35E-07	8.83E+05	8.82E-07	0.65622	0.99995	0.99423

30	R0	C0	R1	C1	R2	Q	a	R^2	R^2 phi
1	2.94E+04	1.16E-07	4.85E+05	6.88E-08	3.23E+05	7.12E-07	0.69533	0.99844	0.93978
2	4.06E+04	6.51E-08	6.92E+04	8.21E-08	1.73E+05	1.00E-06	0.62355	0.99997	0.99911
3	2.88E+02	5.77E-09	2.62E+04	9.43E-07	8.10E+04	1.09E-07	0.84781	0.99978	0.99925
4	8.85E+03	1.49E-07	7.55E+04	1.11E-07	9.82E+05	2.88E-07	0.75144	0.9996	0.99737
5	3.48E+04	1.03E-07	2.11E+05	9.07E-08	7.84E+03	9.49E-07	0.68455	0.99974	0.98494
6	2.80E+04	1.21E-07	1.26E+05	8.81E-08	4.62E+05	1.00E-06	0.64608	0.99993	0.99076

40	R0	C0	R1	C1	R2	Q	a	R^2	R^2 phi
1	3.72E+04	9.96E-08	5.27E+05	7.86E-08	2.60E+05	7.35E-07	0.69063	0.99997	0.99007
2	6.91E+03	1.60E-07	6.90E+04	5.43E-08	6.55E+05	9.95E-07	0.61775	0.9997	0.99466
3	1.32E+02	1.07E-09	2.13E+04	9.94E-07	8.95E+04	1.21E-07	0.83518	0.99988	0.9983
4	1.45E+03	4.18E-09	4.27E+04	1.11E-07	8.22E+05	2.24E-07	0.75122	0.99991	0.96195
5	3.35E+04	9.68E-08	2.03E+05	9.03E-08	6.88E+03	9.57E-07	0.68333	0.99991	0.9942
6	2.34E+04	1.18E-07	1.73E+05	7.80E-08	9.92E+04	9.21E-07	0.65573	0.99994	0.99099

50	R0	C0	R1	C1	R2	Q	a	R^2	R^2 phi
1	2.64E+04	1.55E-07	5.21E+05	6.75E-08	6.90E+04	4.97E-07	0.72856	0.99996	0.98975
2	6.35E+04	5.27E-08	1.03E+05	1.07E-07	5.10E+04	9.46E-07	0.62537	0.9996	0.99531
3	3.33E+02	8.58E-08	1.67E+04	1.00E-06	6.20E+04	1.16E-07	0.84348	0.99974	0.99921
4	1.08E+04	1.18E-07	1.30E+05	7.76E-08	9.90E+05	5.61E-07	0.69869	0.99953	0.99773
5	1.61E+04	1.05E-07	1.93E+05	6.88E-08	3.06E+03	9.12E-07	0.6873	0.99989	0.99686
6	8.93E+04	6.41E-08	3.13E+05	2.08E-07	4.74E+04	8.02E-07	0.66984	0.9999	0.99272

60	R0	C0	R1	C1	R2	Q	a	R^2	R^2 phi
1	1.80E+04	1.74E-07	3.66E+05	8.06E-08	1.88E+05	3.85E-07	0.74703	0.99993	0.98284
2	5.05E+04	5.27E-08	7.86E+03	4.37E-07	1.51E+05	7.54E-07	0.63579	0.99964	0.99558
3	4.29E+02	1.00E-09	1.14E+05	9.67E-07	8.64E+04	9.22E-08	0.86716	0.99975	0.99935
4	1.04E+04	1.30E-07	1.07E+05	8.71E-08	9.26E+05	4.15E-07	0.72223	0.99979	0.99769
5	6.60E+04	1.12E-07	2.25E+05	2.23E-07	4.34E+04	6.11E-07	0.71013	0.99978	0.99326
6	1.83E+04	1.31E-07	1.52E+05	7.57E-08	1.50E+05	1.00E-06	0.64945	0.99977	0.99491
